# Peer support for carers and patients with inflammatory bowel disease: a systematic review

**DOI:** 10.1186/s13643-022-02064-6

**Published:** 2022-09-12

**Authors:** Ada Adriano, Dean M. Thompson, Christel McMullan, Malcolm Price, David Moore, Lesley Booth, Jonathan Mathers

**Affiliations:** 1 Institute of Applied Health Research, University of Birmingham, Birmingham, UK; 2 Centre for Patient Reported Outcomes Research, Institute of Applied Health Research, University of Birmingham, Birmingham, UK; 3 Bowel Research UK, London, UK

**Keywords:** Peer support, Systematic review, Effectiveness, Inflammatory bowel disease

## Abstract

**Background:**

The support provided by people with the same condition, including inflammatory bowel diseases (IBD), has the potential to improve a range of psychosocial outcomes by allowing people with the disease to receive emotional support as well as to learn coping strategies from more experienced peers. The aim of this systematic review was to summarise the evidence on peer support interventions and their effectiveness on people with IBD.

**Methods:**

Bibliographic databases, conference proceedings, grey literature, and clinical trial registers were searched from inception to November 2021. Comparative and single-arm studies that evaluated interventions that were solely or contained in part peer support, for people with IBD and/or their carers of any age and in any setting were included. Effectiveness was evaluated using outcomes relating to physical and psychosocial function, disease control and healthcare utilisation. Data for each outcome were tabulated and presented in a narrative synthesis. Study design specific tools were used to assess risk of bias. Study selection and risk of bias assessment were undertaken by two reviewers independently.

**Results:**

Fourteen completed studies and five ongoing studies met the inclusion criteria. Substantial heterogeneity was observed in the studies in relation to the intervention type and peer support was usually part of a wider intervention. All but one study analysed the total effect of the intervention, so it was not possible to fully isolate the effect of the peer support alone. The appropriateness of outcomes and outcome measurement tools for the assessment of effects was a further key issue. As such, overall, no significant evidence of beneficial effects of peer support interventions on quality of life and other psychosocial outcomes was found.

**Conclusions:**

New randomised controlled trials designed to isolate the effects of peer support are needed to evaluate the (net) effects of peer support only. Agreement on the outcomes to be targeted, and the choice of reliable and validated measurement tools for standalone peer support interventions would provide a focus for further intervention design and evaluation.

**Systematic review registration:**

The protocol was accepted in the international prospective register of systematic reviews (PROSPERO CRD42020168817).

**Supplementary Information:**

The online version contains supplementary material available at 10.1186/s13643-022-02064-6.

## Introduction

Inflammatory bowel disease (IBD) is a group of chronic diseases of the gastrointestinal tract, of which Crohn’s disease (CD) and ulcerative colitis (UC) are the most common types [1]. Genetic and environmental factors (e.g. smoking, stress, diet) as well as immune response play a major role in the pathogenesis of these conditions, although they are not yet fully understood [2].

IBD is prevalent in Europe (ulcerative colitis: 505 per 100,000 in Norway; Crohn’s disease: 322 per 100,000 in Germany) and North America (ulcerative colitis: 286 per 100,000 in the USA; Crohn’s disease: 319 per 100,000 in Canada), with the incidence rising in newly industrialised countries [3]. Early onset IBD in childhood or adolescence has been estimated to occur in approximately 25% of the cases [4]. These diseases are characterised by periods of remission with no or very mild symptoms, alternating with relapses or flare-ups which consist of more active symptoms that occur with unpredictable frequency [5].

People with IBD may experience intestinal symptoms such as abdominal pain, frequent bowel movements, and diarrhoea, as well as extraintestinal symptoms including fatigue and arthralgia [6]. As many as 39% of people with IBD also experience irritable bowel syndrome (IBS), characterised by chronic and recurrent abdominal pain and altered bowel habit [7, 8]. Complications of IBD include ulceration, perforation or obstruction of the intestine requiring surgery. Extra-intestinal manifestations of IBD such as arthritis can also occur [9, 10]. IBD can lead to repeated absence from work or school [11], cause embarrassment and impact on intimacy and social life [12]. This often results in people with IBD experiencing stigma from peers and healthcare professionals and internalising stigma to the detriment of self-care, essential in IBD management [13]. Also, people with IBD may find it difficult to discuss these challenges with people who do not have direct experience with the disease [14]. As a result, these conditions can considerably affect quality of life and psychosocial well-being [15]. High rates of anxiety and depression have been found in people with IBD [16, 17]. Furthermore, psychological factors have been shown to influence disease activity and are associated with frequency of relapses [18, 19].

Peer support interventions are one potential means to provide support to people with IBD. Peer support in the healthcare context has been defined as the emotional, appraisal, and informational assistance provided by people who have experiential knowledge of a specific condition and similar characteristics to the target population, to complement professional health services by sharing personal experiences in relation to a health-related issue [20].

Peer support can be offered through multiple modes of delivery, including collectively within groups or individually one-to-one, through face-to-face or digital routes via the Internet or phone [21, 22]. Peer support interventions have the potential to empower people through learning coping strategies and acquiring self-management skills, enhancing well-being and self-esteem [21]. They have been implemented and evaluated in a range of chronic conditions, with evidence suggesting that they can impact a range of self-management, disease control, and psychosocial outcomes. For example, peer support has been shown to have a favourable effect in improving glycaemic control in patients with type 2 diabetes [23]. In addition, people with depression participating in peer support programmes reported greater reduction in mean depression scores when compared to usual care [24].

A scoping search was undertaken in October 2019 in Epistemonikos and EMBASE, using free and index terms, where possible, relating to peer support and IBD. The search identified no systematic reviews evaluating peer support interventions for people with IBD. In the absence of such a review, the composition and effectiveness of IBD peer support interventions and the outcomes that might be targeted by these interventions remain unclear. Furthermore, gaps in the primary research literature, and information about the quality of existing primary research on peer support for IBD, require elucidation to inform future research [25].

Therefore, this systematic review aims to summarise and critically analyse the evidence relating to the following questions:

- What peer support interventions have been researched in people with IBD; what are the characteristics of peers and people with IBD?
- What is the effectiveness of peer support interventions in people with IBD?

## Methods

This systematic review has been reported according to the Preferred Reporting Items of Systematic Reviews and Meta-Analysis (PRISMA) guidelines (see Supplementary Table 1 for the PRISMA checklist) [26]. The protocol was accepted in the international prospective register of systematic reviews (PROSPERO CRD42020168817) [27].

## Search strategy

The following databases were searched:

- Bibliographic databases: MEDLINE, EMBASE, Cochrane CENTRAL, CINAHL, PsycINFO;
- Conference proceedings: Conference Proceedings Citation Index (via Web of Science);
- Dissertation and theses: ProQuest;
- Grey literature: Open grey (https://opengrey.eu).

The databases were searched from inception to December 2019, and updated on 12 November 2021 using index and free terms for IBD (as well as its different subtypes) and peer support. A detailed search strategy was developed for MEDLINE and adapted for each database (Supplementary Table 2). No language restrictions were applied. Trial registries (ClinicalTrials.gov, WHO International Clinical Trial Registry Platform (ICTRP) and International Standard Randomised Controlled Trial Number (ISRCTN) registry) were searched for ongoing trials. Reference lists of included studies and relevant reviews identified at the screening stage were checked to identify further eligible studies.

## Selection criteria and study selection

Study selection criteria were:

### Population

People of any age in any setting diagnosed with IBD as well as carers of people with IBD. Studies on a broader population (e.g. including people with other chronic conditions) were included but considered for analysis only if data specific for the population with IBD were presented as a subgroup or could be extracted.

### Intervention

Peers were defined as someone with experiential knowledge of IBD, as a person with the condition or as a carer of a person with the condition, that provides support in any setting, through any mode of delivery (e.g. group, one to one, face to face, web-based, computer-based, via telephone) to people with IBD or carers of people with IBD.

Peer support could constitute the totality of the intervention or could be one component of a multi-component intervention. In the latter case, studies were included when the peer support was one of the hypothesised intents of the intervention (i.e. this may include people with IBD being deliberately placed in an environment in which they are encouraged to discuss their experiences of living with IBD—the peer support is intentional, the content of which may be organised or spontaneous). Conversely, studies in which peers could engage in an incidental manner without intent (i.e. people with IBD having the opportunity to talk through being co-located in the context of a programme aimed at providing professional support) were excluded.

### Comparator

Any intervention including different types of peer support, or no intervention (e.g. wait-list group).

### Outcomes

Outcomes relating to:

- Disease control (e.g. disease activity and remission, overall survival, occurrence of complications, change in bowel symptoms, pain or discomfort);
- Physical and psychosocial function, and quality of life (e.g. Health-related quality of life, energy and fatigue, self-esteem, well-being, social functioning, anxiety, depression);
- Healthcare utilisation outcomes (e.g. Time spent in hospital, medication use).

### Study design

Comparative studies (i.e. randomised controlled trials—RCTs—controlled trials and observational studies) and single-arm studies were included. With regard to the single-arm studies, only before-and-after studies and interrupted time series were considered for the analysis and only those addressing types of intervention, people with IBD, length of follow-up or measuring outcomes not covered by the comparative studies. Both comparative and single-arm studies were assessed for the characteristics of the interventions, peers and people with IBD.

All titles and abstracts were screened for relevance. Articles relevant to the review questions were obtained and assessed for inclusion against the full set of selection criteria. Reasons for exclusion of articles at this stage were documented. Study selection was independently undertaken by two reviewers (AA and CM or DT or JM) with any disagreements being resolved through discussion or, if necessary, referral to a third reviewer (DM).

## Data extraction

Data extraction was carried out by one reviewer (AA), using a piloted data extraction form in Microsoft Word (then collated in Microsoft Excel), while a second reviewer (CM or DT or JM) checked the data extracted for accuracy and completeness [28]. Disagreements were resolved through discussion or, if necessary, referral to a third reviewer (DM). Extracted data included:

- Study characteristics: study design, aim and setting, inclusion and exclusion criteria;
- Study participants: number of participants, age, gender, type of IBD, disease severity and activity, disease duration;
- Intervention and comparator details: details of the peers, details of the support intervention, mode of delivery, frequency.
- Outcomes: outcome measurement tool, tool scale, outcome data for each group, effect size, and measure of uncertainty. If any crossover trials were identified, results from phase I only were extracted and used in the analysis.

Attempts were made to contact trial authors to request missing data.

## Risk of bias assessment

Study design specific quality assessment tools were used:

- Controlled trials: Cochrane Collaboration’s Risk of Bias tool [29]. The quality domains relating to randomisation and allocation concealment were not considered for non-randomised trials.
- Observational studies: Newcastle–Ottawa quality assessment form [30].
- Before-and-after studies: National Heart, Lung and Blood Institute (NIH) Study Quality Assessment Tools [31].
- Interrupted time series: Cochrane Effective Practice and Organisation of Care (EPOC) quality checklist [32].

Quality assessment was independently undertaken by two reviewers (AA and CM or DT or JM) with any disagreements being resolved through discussion or, if necessary, referral to a third reviewer (DM).

## Analysis

Included studies were grouped by study design, population, intervention (type of peer support and mode of delivery), comparator, and outcome. Details on the population, intervention, and any model underpinning the intervention were narratively reported. Data for each of the outcomes were tabulated and presented in narrative synthesis.

In the case of continuous outcomes, the mean difference between study groups (accounting for baseline scores) and the mean difference between before and after-study values, along with 95% CI, are reported in the review for comparative and single-arm studies, respectively. If not reported in the articles and where possible, the between-group difference of the mean score changes from baseline was calculated. In case of insufficient data, *p* values relating to the treatment effect are presented in the review, as reported in the articles.

If relevant dichotomous outcomes were encountered, risk ratio, odds ratio, or rate ratio, depending on data availability, are reported in the review along with 95% CI.

Where possible, evidence was assessed in relation to short, intermediate and long-term effects of the interventions. Data were categorised into the following follow-up period groups: ≤ 3 months, > 3 months, and ≤ 1 year, and > 1 year.

## Results

The search strategy identified 5013 records. After removing duplicates, 3580 titles and abstracts were screened for relevance and 3502 records were subsequently excluded. Seventy-five articles underwent full-text selection (full-text was not available for further 3 articles). Fifty-three records were excluded for the reasons indicated in Supplementary Table 3; 4 conference abstracts were excluded as it was not possible to determine whether the intervention included a peer support component.

**Fig. 1:**
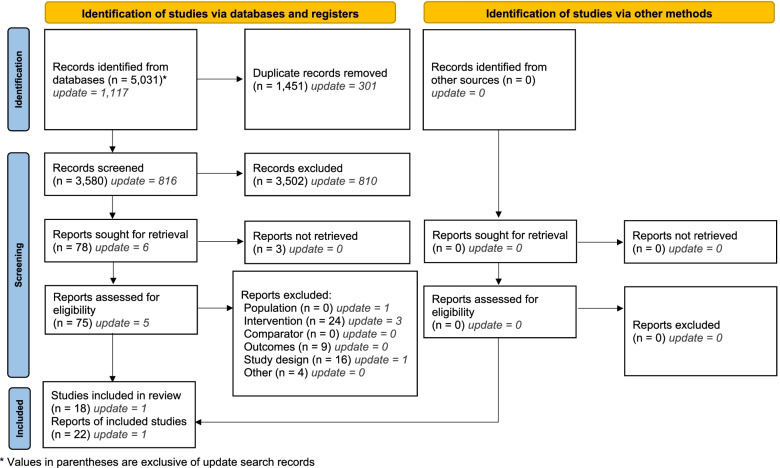
PRISMA flowchart of study selection process *From*: Page MJ, McKenzie JE, Bossuyt PM, Boutron I, Hoffmann TC, Mulrow CD, et al. The PRISMA 2020 statement: an updated guideline for reporting systematic reviews. BMJ. 2021;372:n71. 10.1136/bmj.n71. For more information, visit: http://www.prisma-statement.org/

After updating the search, a further 1117 records were identified. After removing 299 duplicates, 1 additional completed study was identified [33]. Twenty-two articles were included in the review, corresponding to 18 unique studies. These comprised 13 completed studies (11 full-text articles and 2 conference abstracts) and 5 ongoing studies, described in either trial registers or conference abstracts. The PRISMA flowchart of the study selection process is shown in Fig. 1.

## Study characteristics

Of the completed studies, five RCTs [33–37], two controlled studies without randomisation [38, 39], and seven single-arm studies were included [40–46], and these enrolled a total of 2077 participants.

The interventions were delivered in IBD centres as either outpatient or inpatient service (*n* = 8) [33–37, 40, 46], in a local community centre (*n* = 1) [44], in summer residential camps (*n* = 3) [41, 42, 45], within university settings (*n* = 1) [39] or the specific setting was not specified (*n* = 1) [44]. All were based in high-income (European countries, USA, New Zealand) or upper middle-income countries (Brazil, Chile, and China). Interventions lasted between 1 week and 19 months. The study characteristics are described in Table 1.

**Table 1:** Study characteristics

**Study ID**	**Population (disease duration, mean [SD])**	Age mean **(range or SD)**	**Sample size**	**Intervention Components**	**Settings/** **mode of delivery, structure**	**Comparator**	**Outcomes**	**Study design**
**Group-based interventions:** **Patient education + peer support**								
**Berding 2017 **[34]	Outpatients with long-lasting IBD (Intervention group = 10.9 years [10.8]; Waitlist group = 9.6 years [8.9])	Intervention 39.6 (13.2) Control 40.1 (12.3)	207	Education programme covering both medical and psychological aspects Medical sessions: held by physician specialists providing information on IBD (e.g. anatomy, epidemiology, clinical aspects, diagnosis and therapy) Psychological sessions: held by psychologists using patient-centred approach, with participants being encouraged to exchange experiences, ask questions and present individual coping strategies and self-management skills	IBD referral centres/group-based, 1 weekend, 15 participants per batch (16 batches), medical modules lasted 8 h, psychological modules 3.5 h	Waiting group	HRQoL, anxiety, depression, disease activity	RCT
**Reusch 2016 **[35]	Inpatient rehabilitants with IBD (12.8 years [10.5])	43.4 (11.0)	540	Education programme covering medical and psychological modules Medical modules: delivered to large open groups in lecture format by gastroenterologists, providing information on IBD (e.g. anatomy, diagnostic, treatment options). Patients had time to ask questions Psychological modules: delivered in small, closed groups by psychologists using patient-centred approach designed to encourage participants to share their personal experiences about how to best cope with the disease. Patients discussed ways of coping with feelings of anxiety and role-played self-confident communicative behaviour in common difficult social situations	Rehabilitation centres/group-based, eight modules of 1.5 h each, with five medical modules and three psychological modules	Education programme with same medical modules Psychological modules were lecture-based (no interactive approach)	HRQoL, anxiety, depression	Cluster RCT
**Oxelmark 2007 **[36]	People from IBD-outpatient clinic (Intervention group = 4.6 years, range = 1–11); control group = 5.2 years, range = 1–10	Intervention 36.3 (18–71) Control 38.5 (21–59)	46	Group therapy held in unstructured way, but with a certain guidance and special theme to start every session (psychological reactions, receiving information of the diagnosis, coping). The group members had the chance to express their reactions and emotions The medical social worker and psychotherapist took notes during the sessions, which were discussed at the next group therapy session The lectures comprised information and education about the diseases and included time for questions and discussions	IBD-outpatient clinic/group-based, 9 weekly sessions for 3 months circa, lasting 1/2 h	On demand medical and psychosocial/psychological treatment	HRQoL	RCT
**Oliveira 2007 **[37]	People with IBD Supported group = 108.7 months [71.5]	Median (range) Intervention 44.5 (19–63) Control 38 (18–53)	39	Support group delivered by health professionals experienced in dealing with groups, aimed at facilitating and stimulating discussion about the problems and concerns of patients with IBD (e.g. ostomies, surgery, relation to cancer, diet). The meetings aimed mainly to place individuals who shared the same concerns and difficulties side by side. The meetings provided information on the rights of patients with chronic diseases and debated issues related to IBD that could be of interest to the patients	Primary Health care Unit/group-based, programme run on a monthly basis for about 18 months	Regular treatment	HRQoL	RCT
**Krause 2003 **[39]	People with IBD, members of an existing self-help group (Disease duration not reported)	(25–45)	38	Programme aimed at (1) promoting sharing of experiences, emotions, and information regarding the illness, (2) providing information about the psychosocial processes associated with the disease, (3) providing training on mutual social support strategies and coping with stressful events, (4) providing information about the illness	One day per month lasting 2.5 h	Control group not participating to any group or equivalent activity	Quality of life	Non-randomised controlled trial
**Haapamäki 2018 **[40]	Inpatients with IBD (7.9 years, range = 1–37)	43.4 (21–65)	195	Adaptation courses aiming at reducing the impact of the illness on the patient’s working capacity and their mental, physical, and social functioning Participants are provided with adequate information on the disease and specialist support Peer support aimed at encouraging towards a healthy lifestyle and adequate physical exercise	Rehabilitation centres/group-based, SII adaptation courses: 10–12 days, divided into two periods separated by 4–6 months Patient organisation’s adaptation courses: shorter in duration (usually 5 days in one period)	NA	HRQoL, depression	Before-and-after study (observational)
**Szigethy 2009 **[43]**)**	Adolescent girls with IBD + their mothers (Disease duration not reported)	14.5 (2)	12	Education programme (topics included: exercise, diet, stress management, intimacy) concluding with a question-and-answer period facilitated by group leaders/gastroenterologist. Mothers and daughters socialized over dinner for the first hour of each group meeting. Girls and mothers were then separated to allow each group to ask questions independently to the guest speaker, group leaders, and each other. This was followed by discussion between mothers and daughters	Local community centre/group-based, 10 months	NA	HRQoL	Before-and-after study
**Arenas 2018** **(CA) **[44]**)**	Adolescents with IBD (Disease duration not reported)	(13–17)	8	Multidisciplinary programme delivered by 2 paediatric psychologists and a paediatric dietitian, and focused on emotional issues and nutritional aspects	NR/group-based, 6 weekly sessions	NA	Anxiety	Before-and-after study
**Group-based interventions:** **Self-management programme**								
**McDonnell 2014 + Forry 2013 **[38, 47, 48]	People with IBD with no active flare (Disease duration not reported)	NR	53	Self-management programme aimed at enhancing participant self-efficacy through the use of weekly action planning and feedback, modelling of behaviours, group problem-solving and a range of cognitive strategies. Opportunity to meet others with the same condition as themselves. Some co-facilitators were people with the disease to increase the level of empathy and rapport building between the group members	Tertiary referral teaching hospital/group-based, weekly session for 6 weeks, lasting 2.5 h	Waiting group	HRQoL, anxiety, depression	Non-randomised controlled trial
**Zhang 2020 **[33]	Inflammatory bowel disease arthritis (IBDA) (Disease duration not reported)	Routine treatment: 35.48 (4.96) Narrative education: 37.22 (5.34) Peer support 36.85 (4.58) Combined narrative education and peer support: 38.46 (6.18)	132	Patients participated in online discussion groups (WeChat). Patients discussed and shared diseases, treatment and daily life, and comfort and ‘helped each other’. The online group contained doctors, nurses, psychotherapists, and nutritionists	Patients’ questions were gathered and answered twice a week. The program lasted 6 weeks	Routine education	HADS, Polysomnography, Arthralgia numerical rating scale (0–10), Irritable Bowel Syndrome (y/n), Inflammatory factor serum levels	RCT
**Group-based interventions:** **Psychosocial support**								
**Shepanski 2005 **[41]**)**	Children and adolescents with IBD (Disease duration not reported)	(9–16)	61	Summer camp with children of the same age living together along with several counsellors who were trained with information about IBD to understand their needs Children participated in group activities but not in formal IBD educational classes or group sessions to discuss their experiences Informal conversations and sharing experiences among the campers and counsellors about their own illness were common	Summer campgrounds/group-based, 1 week	NA	HRQoL, anxiety	Before-and-after study (observational)
**Plevinsky 2014 + Plevinsky 2012 **[42, 49]	Children and adolescents with IBD (Disease duration not reported)	15.33 (1.07)	25	*Children camp* Campers participating in fun activities ranging from arts and crafts to sports. Camp was staffed with volunteer counsellors *Facebook group* Aimed at facilitating the continuation of the social interaction fostered by the camp experience. Participants were free to like, comment on, or create an unlimited number of original posts within the group	Summer campgrounds/group-based, 1 week (camp) + at least 2 months (Facebook group)	NA	HRQoL	Before-and-after study
**Day 2016** **(CA) **[45]	Children and adolescents with IBD (Disease duration not reported)	Median (range) 14 (10–18)	44	Campers undertaking a range of physical activities and group activities. No specific IBD-related educational activities were included. Children were supervised by volunteer leaders, many of whom also had IBD Benefits thought to derive from mixing with people who are knowledgeable about the illness	Camp/group-based, 4 days	NA	HRQoL	Before-and-after study
**1:1 interventions:** **In-patient peer support**								
**Hashash 2016 + Regueiro 2016 (CA) **[46, 50]	Inpatients with IBD (Disease duration not reported)	NR	677	IBD connect programme delivered by trained volunteer peer specialists that provide the patient and family with support and encouragement to reduce stress and fears, as well as educational materials that are individually tailored for the patient and their family. It serves as a channel to link patients to resources and the services	Hospital-inpatient IBD service/1:1 programme	NA	Stress related to hospitalisation	Before-and-after study (observational)

*Abbreviations*: *CA* conference abstract, *HADS* Hospital Anxiety and Depression Scale, *HRQoL* health-related quality of life, *IBD* inflammatory bowel disease, *NA* not applicable, *NR* not reported, *RCT* randomised controlled trial

1) What peer support interventions have been researched in people with inflammatory bowel disease (IBD); what are the characteristics of the interventions, peers and people with IBD?

## Population characteristics

Seven studies were conducted on adults [33–37, 39, 40], with mean age ranging from 36.3 to 43.9 years (range 18–71) (mean not specified or provided as median in two studies). One study did not report aggregated population characteristic data [33]. Five studies were intended for a younger population (age range 9–18 years and mean range 14.5–15.33 years—mean not specified or provided as median in three studies) [41–45]. With the exception of two studies, a higher percentage of females participated in the studies (range 46–82%) compared to males, and only adolescent girls were recruited in one study [43]. Two studies did not report information on age or gender [38, 46]. Mean disease duration across studies ranged between 4.6 and 12.8 years. Peer characteristics were consistently described across studies as being the ‘same as that of the participants or patients’ (i.e., people with IBD) without separate comprehensive detail of demographic and clinical characteristics.

The percentage of participants with a diagnosis of Crohn’s disease ranged from 28 to 92% (with one study reporting a range of 20–30% across 4 groups of participants). One study comprised participants with inflammatory bowel disease arthritis (IBDA) [33]. The remainder of the participants had ulcerative colitis and, in four studies, IBD type was unclassified for a small proportion of participants (2.1– 5%) [35, 40, 42, 46]. Information on type of IBD was not available in two studies [38, 39]. All participants were in remission, not experiencing active flares or only having mild disease activity in six studies [34–36, 38, 40, 45]. The definition of disease activity and severity was based on different indexes such as the Harvey-Bradshaw index (HBI) and Mayo score for Crohn’s disease and ulcerative colitis participants respectively, and the German Inflammatory Bowel Disease Activity Index (GIBDI). In one study, participants were inpatients but their disease status was not specified [46]. The remainder of the studies did not provide information regarding disease activity or severity.

## Intervention characteristics

### Group-based interventions

Thirteen studies described group peer support that was mutually provided by participants, during the course of ad hoc face-to-face discussion and Q&A sessions, an online mutual assistance group, or as a result of spontaneous interactions, according to their experience with specific aspects of the condition (no training was received by the peers) [33–45, 51–53]. In 12 of these studies, peer support was only one component of multi-component interventions delivered by healthcare professionals (i.e. gastroenterologists and/or dietitians and/or nurses and/or psychologists) that were meant to provide professional support or guide the group through the peer support sessions with minimal involvement in the discussions. One of these studies compared peer support with education, peer support and education combined, and treatment as usual [33]. In two studies, the programmes were co-led by peers who had personal experience with the disease [37, 45].

These group-based interventions aimed at giving participants the opportunity to express their emotions and share their feelings with peers using an interactive approach [36, 39, 44]. Participants were encouraged to share their experiential knowledge of IBD with other participants focusing on coping strategies and self-management skills [33–35, 37, 39, 40, 43]. In Zhang (2020), the online peer support group was supported by doctors, nurses, psychotherapists, and nutritionists, who gathered and answered patients’ questions twice a week [33]. In Berding (2017) and Reusch (2016), the interventions included ‘psychological’ modules that differed from ‘medical’ modules that were also provided [34, 35]. The ‘psychological’ modules took a patient-centred approach that enabled participants to engage in various tasks and discussions where the newly diagnosed could learn from the more experienced (e.g. coping with anxiety, self-confident communicative behaviour in common difficult social situations). Peer support offered in specifically designed adaptation courses contributed to the overall aim of encouraging a healthy lifestyle through group activities and social programmes [40].

In nine studies, the intervention programme also comprised an educational component with information about the clinical aspects of the illness, its epidemiology, pathogenesis and therapy being offered by professionals in the form of a lecture [33–37, 39, 40, 43, 44].

In McDonnell (2014), the programme focused on the concept of self-management through weekly action planning and feedback. Using cognitive strategies, it aimed to improve self-management in an empathic environment using peer facilitators with IBD [38].

In three out of the five studies intended for children and adolescents, peer support was provided in the context of summer camps, where participants engaged in various fun group activities (e.g. sport, dance, cooking, adventure-based activities) without any formal educational or psychotherapeutic sessions [41, 42, 45]. Informal conversation and spontaneous interaction among children were expected to occur, and were believed to help children to exchange personal experiences with IBD and in doing so learn coping strategies and improve self-esteem. One of these camps was also followed by the creation of a Facebook group where camp participants could continue the social interaction within the group. Investigators facilitated the online interaction by posting IBD-related questions.

### One-to-one peer support

One study described a volunteer peer specialist programme incorporated into an inpatient service [46]. Peers were trained volunteers, individually matched to patients based on age, gender, and disease experience. Peers offered one-to-one emotional support to patients and their family in their hospital room by sharing their experiences, addressing patients’ concerns, and providing tailored educational materials. Unlike the group-based studies, this intervention did not include any other ‘non-peer support’ components.

## Comparator characteristics

In five out of seven controlled studies, the comparator group did not engage in any alternative activities or received the same intervention later during the course of the study [34, 36–39]. In Reusch (2016), instead, the comparator included medical sessions that were identical to those of the intervention [35]. However, the psychological modules were delivered in a lecture-based format as opposed to the more interactive sessions delivered in the intervention group. In Zhang (2020), peer support was compared with education, peer support and education combined, and treatment as usual [33]. Details on the population, interventions, and comparators can be found in Table 1.

2) What is the effectiveness of peer support interventions in people with inflammatory bowel disease?

## Risk of bias assessment

Substantial heterogeneity relating to type of intervention, comparators, and outcome measurement tools was noted in the included studies and precluded the possibility of undertaking meta-analysis. Risk of bias assessment is detailed in the Supplementary Table 4.

The main limitation of included studies was the lack of blinding of participants, especially considering the use of patient-reported outcomes, although this may be due to the difficulty of designing a sham peer support intervention. In seven studies (3 controlled and 4 single-arm), another common quality concern was data completeness with a high proportion of study participants not completing questionnaires (up to 88.6%) [34, 37, 38, 40, 41, 45, 46]. Whilst in one study, no differences in participants characteristics were observed between participants completing the study and those whose outcome data were not available [34], in the remainder of the studies no reasons for dropout were provided and risk of attrition bias cannot be excluded.

With regard to the controlled studies, two studies used appropriate methods of allocation concealment [34, 35], although details on the randomisation sequence method were not given in one study [35]. Two studies did not describe the randomisation method [36, 37]. No randomisation occurred in one study which used convenience samples [38]. In another study, participants in the two groups did not seem to be recruited from the same source population [39].

## Effectiveness of peer support interventions

A narrative synthesis is presented below, and results are also shown in Supplementary Tables 5–11. Meta-analysis was not feasible for any of the outcomes considered, owing to heterogeneity in type of intervention, comparators, measurement tools and data availability.

### Health-related quality of life

Eleven studies measured the effects of peer support interventions on health-related quality of life (HRQoL) [34–43, 45]. Eight of these studies used disease-specific quality of life measurement tools such as IMPACT II or III (*n* = 4), Inflammatory Bowel Disease Questionnaire (IBDQ) (*n* = 2) and Short Inflammatory Bowel Disease Questionnaire (SIBDQ) (*n* = 2). Generic questionnaires evaluating HRQoL were chosen in five studies, namely SF-36 (*n* = 2), the short from SF-12 (*n* = 2) and 15-D (*n* = 1).

### Randomised controlled trials (RCTs)

In the four RCTs [34–37], the mean difference between study groups accounting for baseline imbalances was not reported, nor could it be calculated due to lack of available data.

An increase in HRQoL was observed at 3 months and 6 months, with a slight decrease at 12 months both in the intervention group and control groups but the difference between the groups was not statistically significant.

### Non-randomised controlled studies

Neither of the two non-randomised studies reported the mean difference between study groups (nor could it be calculated due to lack of available data) [38, 39]. In Krause (2003), the authors found statistically significant difference between the study groups at the end of the intervention (1 year) for the intestinal domain of the SIBDQ questionnaire (*p* = 0.030) [39]. However, the baseline data were not provided to judge any imbalances at baseline that could contribute to results.

### Before-and-after studies

In the adaptation courses, improvements from baseline in HRQoL were seen at the end and after the intervention (6 and 12 months). The changes from baseline exceeded the minimal clinically important difference (i.e. 0.015 with questionnaire scale being 0–1) at all time points (end of course, 6 and 12 months) (data provided by the author) [40].

Benefits to quality of life were also observed at the end of the summer camp weeks (mean change from baseline 8.09 [95%CI 3.24, 12.93] and 5.7 [95%CI 0.52, 10.88] (Supplementary Table 6) but not at 2 months after participation to the Facebook group [41, 42].

Mean change from baseline could not be calculated for the remainder of the before-and-after studies.

### Anxiety and depression

Seven studies explored the effects of peer support interventions on anxiety and depression [33–35, 38, 40, 41, 44]. Various self-report questionnaires were used to carry out the evaluation, such as the Patient Health Questionnaire-4 (PHQ-4) (*n* = 2), STAI (State-Trait Anxiety Questionnaire) (*n* = 2), Beck’s Depression Inventory (BDI) (*n* = 1) and Hospital Anxiety and Depression scale (HADS) (*n* = 2).

#### Randomised controlled trials (RCTs)

For the first two RCTs, results from ANCOVA analyses could not be obtained. The differences at follow-up in both anxiety and depression scores between the groups were very small and not statistically significant [34, 35]. In Zhang (2020), Kruskal–Wallis *H* test was used to compare treatment groups (peer support, health education, peer support and health education combined, and routine treatment) [33]. Depression scores of patients receiving combined education and peer support were significantly lower than the other three groups fF/*χ*^2^ = 19.92 (*p* < 0.0001). Depression scores for peer support and health education groups separately were significantly lower compared to routine treatment, though no significant difference was detected between health education and peer support groups.

#### Before-and-after studies

A statistically significant decrease in depression score was shown after the end of the adaptation courses (− 2.85 [95%CI − 2.18, − 3.53]) and it was maintained at 6 and 12 months (− 2.76 [95%CI − 1.58, − 3.95] and − 2.12 [95%CI − 1.03, − 3.21], respectively—data provided by author) [40]. The initial BDI score was 11.8, which is only 1.8 higher than a score indicating no mood disturbances, suggesting a population with lower severity of depression participating in the study.

It was not possible to get full results from the other three studies (one non-randomised controlled study and two before-and-after studies). Descriptive results are provided in Supplementary Table 8.

### Other outcomes

#### Disease activity and severity

One RCT measured disease activity and severity using the German Inflammatory Bowel Disease Activity Index (GIBDI) for both patients with Crohn’s disease and ulcerative colitis [34]. This study found no evidence of difference between the study groups (mean differences were not reported, nor could they be calculated).

Haapamäki (2018) used specific measures for each of the conditions (Mayo score and Harvey-Bradshaw index—HBI) [40]. At baseline both participants with ulcerative colitis and Crohn’s disease had mild disease but were not in remission. A decrease in disease severity was observed at 6 and 12 months for ulcerative colitis participants (1.52 [95%CI 1.06, 1.98] and 1.12 [95%CI 0.56, 1.68]) but not for those with Crohn’s disease (0.30 [95%CI − 1.25, 1.86] and 0.55 [95%CI − 1.51, 2.61]). Also, in the former group, the Mayo score decreased to values indicative of remission [40].

No additional outcomes related to disease control (e.g. complications, change in bowel symptoms, pain or discomfort) have been measured in any of the included studies. However, a reduction in the use of healthcare services such as visits to health professionals and investigations was seen in Haapamäki (2018) (Supplementary Table 11).

#### Psychosocial function

Berding (2017) reported an improvement in the outcomes assessed (IBD concerns, fear of progression, coping with anxiety, coping with the disease) in the intervention group compared to the waitlist group at 2 weeks and 3 months [34]. In particular, better scores at follow-up were achieved in all dimensions of coping including handling of emotions and development of strategies. However, the improvements appeared to be small and their clinical relevance remains unclear [34]. No statistically significant between-group differences were shown in Reusch (2016) for these outcomes [35]. In Oxelmark (2007), no significant differences in sense of coherence between the intervention and control groups were obtained at 6 and 12 months [36].

#### Social connectedness and social support

In one before-and-after study, participants at the summer camp were surveyed regarding social connectedness and social support. The study showed no significant improvement in these outcomes but greater satisfaction with the support received was noted 2 months after children joined the Facebook group (Supplementary Table 11) [42].

#### Knowledge of disease

Two RCTs evaluated participants’ knowledge about coping strategies and medical aspects of the condition as well as impact of such knowledge on their attitudes [34, 35]. Even though an effect in time was observed in the groups receiving peer support, the between-group difference was very small and in Reusch (2016), not statistically significant [34, 35].

A significant increase in knowledge was shown 1 month after a children camp, using a questionnaire specifically addressing children with IBD (IBD-KID), and at the end of the adaptation courses [40, 45]. The effect on participants’ disease knowledge was also confirmed after the end of the intervention at 6 and 12 months.

#### Stress during hospitalisation

Stress during hospitalisation was one of the two outcomes reported by Hashash (2016) and was measured through a survey among the hospitalised people who received peer support (IBD connect programme) [46]. The questions were not validated. Stress decreased on average from 56 to 18% among the 77 patients that responded to the survey [46].

#### Sleep

One RCT measured sleep efficiency (the ratio of total sleep time to bedtime) at the end of the intervention (4–6 weeks) [33]. Quality of sleep was significantly greater for peer support and narrative education combined compared to peer support and narrative education alone, and routine treatment. Only narrative education and narrative education and peer support combined were significantly improved between the beginning and end of intervention.

#### Arthralgia

One RCT measured arthralgia at the end of the intervention (4–6 weeks) [33]. Pain was significantly lower for peer support and narrative education combined compared to peer support and narrative education alone, and routine treatment. There were no significant differences between all other conditions, though pain was significantly reduced between the beginning and end of the interventions.

#### Irritable bowel syndrome

One RCT measured the presence or absence of IBS via medical record review at the end of the intervention (4–6 weeks) [33]. IBS was indicated in significantly fewer participants receiving peer support and narrative education combined after 6 weeks, with no significant differences noted between all other interventions nor between the beginning and end of all other interventions.

## Discussion

This systematic review found no significant and sustained evidence of beneficial effects of interventions that include peer support components for IBD on HRQoL, anxiety, depression and other outcomes related to psychosocial function. Even when a small between-group difference was observed, the possibility that knowledge of group allocation might have inadvertently affected participants’ responses in the questionnaires, particularly in the case of waitlist control groups, resulting in an overestimation of the interventions’ effects, cannot be ruled out. Some of the single-arm studies seemed to suggest a beneficial effect on a limited set of outcomes. However, due to the nature of the study design, a regression to the mean cannot be excluded. Uncertainties also arise over the sustained impact of children’s camps on quality of life, whose effects were only observed immediately after the end of the programme.

The recruitment of a relatively healthy IBD population could explain some of the results (i.e., ceiling effect). In Berding (2017), the HRQoL baseline score is close to that of the general population in Germany (the country where the study was conducted) for both physical and mental domains [54]. In Oxelmark (2007), the mean IBDQ score of participants at baseline was marginally outside of clinical remission. Despite the study eligibility criteria being inclusive, skewed recruitment of individuals in remission who are also committed to enhancing their self-management skills and well-being, might have occurred [36]. As for the before-and-after studies, improvements in quality of life and depression were shown after the intervention in Haapamäki (2018) where participants had, instead, mild disease. In this case, such improvements were accompanied by a decrease in disease severity in people with ulcerative colitis only [40].

It should be noted that, as the included studies on the whole analysed the total effect of multi-component interventions, it was not possible to isolate the effect of the peer support components. Of the multi-component interventions, Reusch compared the addition of a peer support element to the intervention group, but without demonstrating the effectiveness of this [35].

Indeed, possibly one of the key observations from our review is that to date there have been few attempts to evaluate the effectiveness of standalone peer support interventions. One exception in this review is the IBD CONNECT programme [46], targeted at inpatients in an attempt to reduce the stress related to hospitalisation. However, Hashash (2016) utilised a bespoke study-specific outcome measure potentially lacking wider applicability and validation. Also, Zhang (2020) compared peer support, narrative education, and peer support and narrative education combined, though the peer support group also received 2 initial 5-min appointments and were prescribed mesalamine (2.0 g/day) [33].

A systematic review of reviews on peer support across chronic conditions highlighted similar methodological limitations [55]. Syntheses of peer support for cardiovascular disease and diabetes indicated a significant small-medium effect on clinical surrogates including blood pressure and blood glucose. Quality of life and depression were two of the most frequently measured outcomes, though effect sizes were small and not statistically significant. It remains unclear whether these data can be related to IBD. Any effort to develop or refine IBD peer support using successful examples of peer support for other chronic conditions may benefit from an intervention mapping approach; mapping hypothetically effective peer support components onto the needs of people with IBD [51].

The issue of outcome selection and the appropriateness of outcomes for the assessment of the effects of peer support interventions is also a key issue. Our review demonstrates that a broad range of outcomes and measurement tools have been included in studies to date, with little consistency across studies. Categories of outcomes included HRQoL, anxiety and depression, psychosocial function, disease severity and knowledge, and standalone outcomes such as stress during hospitalisation. Perhaps a key area for further reflection is the core target outcomes for peer support interventions and how these should be assessed, as well as the mechanisms by which peer support interventions might realise their effect. As peer support approaches might be theorised to act upon and via psychosocial function and processes, it could be argued that these should be the key foci for assessment, with other categories of outcome (e.g. HRQoL, knowledge, disease severity, and clinical measures) as secondary outcomes.

### Strengths and limitations of the included studies

The included studies showed substantial heterogeneity in relation to the intervention type. Peer support was only one of the components of various multi-component interventions that also aimed at providing professional support and medical knowledge and that differed in settings, frequency, and mode of delivery. The extent to which the interaction among peers contributed to the observed results remains unclear. This review has highlighted that the use and evaluation of concomitant patient education by healthcare professionals alongside peer support is ingrained in the literature. However, peer support intervention components are reported only sparingly. Further peer support research should be reported with depth of the intervention description to enable onward intervention assessment and development, and study designs should be used that allow for assessment of effect of components of multifactorial interventions containing peer support.

The ongoing studies (Table 2) demonstrate a substantial change in mode of peer support provision, with 1:1 mentor–mentee relationships being increasingly used instead of group-based interventions, and remote support via telephone, email, or videocall being preferred to face-to-face contact in most cases. In addition, the interventions being evaluated within ongoing studies are predominantly standalone peer support interventions focused on psychosocial support, as opposed to multi-component education and self-management interventions with a peer support component. Four of the ongoing studies are due to be completed between May 2020 and February 2024 when an update to this systematic review may be warranted.

**Table 2:** Characteristics of ongoing studies

**Study ID**	**Population**	Age **(inclusion criteria)**	**Estimated** **sample size**	**Intervention**	**Comparator**	**Outcome**	**Study design**
**Blockman 2018** **(CA) **[56]	Teens living with chronic illness	12–20^a^	26	Peer support for teens and their parents	NA	Physical health, depression	Before-and-after study
**Luu 2011 **[57]	Individuals with IBD and their family members	NR	NR	Power of two: peer support programme providing psychosocial support services by telephone or email through peer laypersons with experiential knowledge of comparable medical and personal concerns	NA	Anxiety, health practices	Before-and-after study
**NCT03938324 2019 **[58]	Adolescents and young adults with chronic disease	16–22	225	Peer i-coaching: telephone/text-based secure interface to allow adolescents and young adults to access knowledge, experience, and instrumental/emotional support from a trained peer coach (18–26 years), who has already developed independence and is an active self-manager	Monthly newsletter with educational content and monthly phone call from study staff to answer questions regarding content	HRQoL, emotional health	RCT
**NCT03827109 2019 **[59]	Youth with IBD	10–17	300	Mentee-mentor relationships with group educational activities, online educational information, and a parent support component Weekly contact (e.g. text, phone), with in-person contact 1–2 times per month, one of which can be attending a group activity together	Educational group events on the same topics (with no social time), educational information posted online, and monthly encouragement to engage in activities in the community	HRQoL, disease severity, number of hospital admissions, clinic appointments, missed appointments, mentor and parent QoL	RCT
**NCT03630146 2018 **[60]	Teens with IBD	12–18	262	iPeer2Peer programme: peer mentorship programme where mentors will encourage youth to develop and engage in disease self-management skills and provide social support, through Skype video sessions	Standard care but without the iPeer2Peer Program	HRQoL, anxiety, disease activity, health Services	RCT

*Abbreviations*: *CA* conference abstract, *HRQoL* health-related quality of life, *IBD* inflammatory bowel disease, *NA* not applicable, *NR* not reported, *RCT* randomised controlled trial

^a^ Information retrieved from clinicaltrials.gov register (NCT03170167). The study described in the register is conducted on people with various chronic diseases including inflammatory bowel disease

A wide range of outcome measurement tools have been used, which are likely to differ in reliability and sensitivity to change. In addition, poor reporting has been a recurring issue. Relevant information was often missing with respect to the weight attributed to peer support as compared to other elements of the interventions. This led to the exclusion of a number of studies where uncertainties remained. For example, Larsson (2003) [52] evaluated an education programme led by professionals covering general medical information about IBD, treatment options, diet and how to adapt and cope with this chronic condition, allowing time for group discussion. As part of the participants’ evaluation of the programme, meeting other individuals with IBD and exchanging experiences were particularly valued. However, whether the ‘peerness’ of this programme was intentional could not be clarified.

Furthermore, the minimal clinically important difference was rarely reported by authors. Between-group difference (or within-group difference for non-comparative studies) as well as outcome data for each of the groups were often not provided, which resulted in inability to adequately interpret the findings. The representativeness of the study participants may also be questioned, as people who are more health conscious or in greater need of support may be more likely to participate in this kind of intervention, and randomisation was not always conducted or adequately detailed.

### Strengths and limitations of the review

The main strength of this review was the comprehensive and updated search, which was based on multiple index and free terms relating to the condition and interventions, with no temporal or language restriction. Attempts to overcome publication bias were made by searching conference abstracts, grey literature and trial registries. Given the difficulty in identifying grey literature, more than a single database could have been searched. Hand-searching proved essential in ensuring that no relevant studies were missed: the intervention evaluated in Krause (2003), whose record had otherwise been excluded at the screening stage (based on the information reported in the abstract), was detailed in a review identified through checking references of included studies, which led to the final inclusion of the study. Moreover, the eligibility criteria were broad enough to allow inclusion of studies in which peer support did not constitute the sole or main component of the intervention, as these could be valuable in showing additional ways of delivering this form of support.

However, there are also some limitations. Efforts were made to contact authors for clarity on data. For one conference abstract it was not possible to obtain further information [44]. For another conference abstract, the full-text study was published in February (2020) (i.e., after the review’s cut-off date) while missing data had been requested from the study’s author [45]. In addition, due to the heterogeneity observed in both intervention and outcomes, meta-analysis was not feasible. With regard to the single-arm studies, the results provided in the review were based on the mean change from baseline, which could also be explained by the regression to the mean, especially considering that long-term effects were not assessed in most cases. Comparative studies evaluating the same interventions could provide more reliable estimates of effect.

### Recommendation for future research

There is need for new RCTs that evaluates the (net) effects of peer support only through design such as component analysis. However, such an approach should be taken with caution as one mechanism by which peer support is thought to work is by bridging and engaging with other components of services [53]. Moreover, effects on psychosocial function and processes should be the focus of future evaluation, whilst supported by data on quality of life and disease severity/activity.

Consistencies in the choice of reliable and validated measurement tools is key when planning future studies. Effort should also be made to improve study reporting, in particular for treatment effect, statistical analysis, and outcome measures. However, limitations in the assessment of patient-reported outcomes due to lack of participant blinding are anticipated.

## Conclusion

This systematic review has summarised primary research on peer support for IBD and highlighted that the available literature is insufficient to robustly establish effectiveness due to complex concomitant interventions, heterogeneity between study design and types of peer support, and weaknesses in research design and reporting of primary research. At present, there is a lack of good evidence on the effect of peer support interventions on the range of outcomes detailed here. This is partly due to poor study design and quality, but also because of a lack of standalone peer support interventions that target specified psychosocial processes and outcomes that are amenable to evaluation. The lack of standalone peer support interventions means that effectiveness findings here cannot be separated out from concomitant interventions and any attempt to generalise should be contextualised within the wider package of treatment evaluated. The ongoing studies may provide more robust estimates of the effects of targeted peer support for certain groups of IBD patients, including young people. Agreement on the outcomes to be targeted by standalone peer support interventions would provide a focus for further intervention design and evaluation.

## Supplementary Information

**Additional file 1: Supplementary Table 1.** PRISMA 2020 Checklist. **Supplementary Table 2.** Search strategy for MEDLINE. **Supplementary Table 3.** Excluded studies and reason for exclusion. **Supplementary Table 4.** Risk of bias assessment. **Supplementary Table 5.** Health-related quality of life (HRQoL) in RCTs and non-randomised studies. **Supplementary Table 6.** Health-related quality of life (HRQoL) for before-and-after studies. **Supplementary Table 7.** Anxiety and depression in RCTs and non-randomised studies. **Supplementary Table 8.** Anxiety and depression in before-and-after studies. **Supplementary Table 9.** Patient education and knowledge about the illness. **Supplementary Table 10.** Other outcomes measured in RCTs. **Supplementary Table 11.** Other outcomes measured in before-and-after studies.

## Data Availability

Included study data are included in this report’s supplementary information files.

## References

[1] Fakhoury M, Negrulj R, Mooranian A, Al-Salami H (2014). Inflammatory bowel disease: clinical aspects and treatments. J Inflamm Res.

[2] Zhang Y-Z, Li Y-Y (2014). Inflammatory bowel disease: pathogenesis. World J Gastroenterol.

[3] Ng SC, Shi HY, Hamidi N, Underwood FE, Tang W, Benchimol EI (2018). Worldwide incidence and prevalence of inflammatory bowel disease in the 21st century: a systematic review of population-based studies. Lancet.

[4] Shim JO (2019). Recent advance in very early onset inflammatory bowel disease. Pediatr Gastroenterol Hepatol Nutr.

[5] Liverani E, Scaioli E, Digby RJ, Bellanova M, Belluzzi A (2016). How to predict clinical relapse in inflammatory bowel disease patients. World J Gastroenterol.

[6] Perler B, Ungaro R, Baird G, Mallette M, Bright R, Shah S (2019). Presenting symptoms in inflammatory bowel disease: descriptive analysis of a community-based inception cohort. BMC Gastroenterol.

[7] Halpin SJ, Ford AC (2012). Prevalence of symptoms meeting criteria for irritable bowel syndrome in inflammatory bowel disease: systematic review and meta-analysis. Am J Gastroenterol.

[8] Drossman DA, Hasler WL (2016). Rome IV-functional GI disorders: disorders of Gut-Brain interaction. Gastroenterology.

[9] Hendrickson BA, Gokhale R, Cho JH (2002). Clinical aspects and pathophysiology of inflammatory bowel disease. Clin Microbiol Rev.

[10] Ossum AM, Palm Ø, Cvancarova M, Solberg IC, Vatn M, Moum B, Høivik ML; IBSEN study group. Peripheral arthritis in patients with long-term inflammatory bowel disease. Results from 20 years of follow-up in the IBSEN study. Scand J Gastroenterol. 2018;53(10-11):1250-56. 10.1080/00365521.2018.1518482. Epub 2018 Oct 24. PMID: 30353756. 10.1080/00365521.2018.1518482 30353756

[11] Nurmi E, Haapamäki J, Paavilainen E, Rantanen A, Hillilä M, Arkkila P. The burden of inflammatory bowel disease on health care utilization and quality of life. Scand J Gastroenterol. 2013;48(1):51-7. 10.3109/00365521.2012.685750. Epub 2012 May 14. PMID: 22577851. 10.3109/00365521.2012.685750 22577851

[12] Devlen J, Beusterien K, Yen L, Ahmed A, Cheifetz AS, Moss AC (2014). The burden of inflammatory bowel disease: a patient-reported qualitative analysis and development of a conceptual model. Inflamm Bowel Dis.

[13] Taft TH, Keefer L (2016). A systematic review of disease-related stigmatization in patients living with inflammatory bowel disease. Clin Exp Gastroenterol.

[14] Leshem RN. Inflammatory bowel disease support groups: a primer for gastroenterology nurses. Gastroenterol Nurs. 2003;26(6):246-50. 10.1097/00001610-200311000-00006. PMID: 14676612. 10.1097/00001610-200311000-00006 14676612

[15] Ghosh S, Mitchell R (2007). Impact of inflammatory bowel disease on quality of life: results of the European Federation of Crohn's and Ulcerative Colitis Associations (EFCCA) patient survey. J Crohns Colitis.

[16] Mikocka-Walus A, Knowles SR, Keefer L, Graff L. Controversies Revisited: A Systematic Review of the Comorbidity of Depression and Anxiety with Inflammatory Bowel Diseases. Inflamm Bowel Dis. 2016;22(3):752-62. 10.1097/MIB.0000000000000620. PMID: 26841224. 10.1097/MIB.0000000000000620 26841224

[17] Byrne G, Rosenfeld G, Leung Y, Qian H, Raudzus J, Nunez C (2017). Prevalence of anxiety and depression in patients with inflammatory bowel disease. Can J Gastroenterol Hepatol.

[18] Maunder RG, Levenstein S (2008). The role of stress in the development and clinical course of inflammatory bowel disease: epidemiological evidence. Curr Mol Med.

[19] Mittermaier C, Dejaco C, Waldhoer T, Oefferlbauer-Ernst A, Miehsler W, Beier M (2004). Impact of depressive mood on relapse in patients with inflammatory bowel disease: a prospective 18-month follow-up study. Psychosom Med.

[20] Dennis C-L (2003). Peer support within a health care context: a concept analysis. Int J Nurs Stud.

[21] Embuldeniya G, Veinot P, Bell E, Bell M, Nyhof-Young J, Sale JE (2013). The experience and impact of chronic disease peer support interventions: a qualitative synthesis. Patient Educ Couns.

[22] Heisler M (2007). Overview of peer support models to improve diabetes self-management and clinical outcomes. Diabetes Spectrum.

[23] Qi L, Liu Q, Qi X, Wu N, Tang W, Xiong H (2015). Effectiveness of peer support for improving glycaemic control in patients with type 2 diabetes: a meta-analysis of randomized controlled trials. BMC Public Health.

[24] Pfeiffer PN, Heisler M, Piette JD, Rogers MAM, Valenstein M (2011). Efficacy of peer support interventions for depression: a meta-analysis. Gen Hosp Psych.

[25] Chalmers I, Glasziou P (2016). Systematic reviews and research waste. Lancet.

[26] Page MJ, McKenzie JE, Bossuyt PM, Boutron I, Hoffmann TC, Mulrow CD, Shamseer L, Tetzlaff JM, Akl EA, Brennan SE, Chou R, Glanville J, Grimshaw JM, Hróbjartsson A, Lalu MM, Li T, Loder EW, Mayo-Wilson E, McDonald S, McGuinness LA, Moher D (2021). The PRISMA 2020 statement: an updated guideline for reporting systematic reviews. BMJ (Clinical research ed).

[27] Adriano A, McMullan C, Thompson D, Moore D, Mathers J. Peer support interventions in people with inflammatory bowel disease: PROSPERO; 2020. Available from: https://www.crd.york.ac.uk/prospero/display_record.php?ID=CRD42020168817. 10.1186/s13643-022-02064-6 PMC9465919 36096828

[28] Cochrane Effective Practice and Organisation of Care (EPOC) (2019). What study designs can be considered for inclusion in an EPOC review and what should they be called? EPOC resources for review authors.

[29] Higgins JPT, Altman DG, Gøtzsche PC, Jüni P, Moher D, Oxman AD (2011). The Cochrane Collaboration’s tool for assessing risk of bias in randomised trials. Bmj.

[30] Wells GA Shea B OCD, Peterson J, Welch V, Losos M, Tugwell P. . The Newcastle-Ottawa Scale (NOS) for assessing the quality of nonrandomised studies in meta-analyses. Unknown. Available from: http://www.ohri.ca/programs/clinical_epidemiology/oxford.asp. Cited 2020 26 March.

[31] National Heart L, and Blood Institute, National Institutes of Health (NIH). Quality assessment tool for before-after (pre-post) studies with no control group unknown. Available from: https://www.nhlbi.nih.gov/health-topics/study-quality-assessment-tools. Cited 2020 26 March.

[32] (EPOC). CEPaOoC. EPOC Resources for review authors. Suggested risk of bias criteria for EPOC reviews 2017. Available from: https://epoc.cochrane.org/resources/epoc-resources-review-authors. Cited 2020 26 March.

[33] Zhang Y, Pi B, Xu X, Li Y, Chen X, Yang N (2020). Influence of narrative medicine-based health education combined with an online patient mutual assistance group on the health of patients with inflammatory bowel disease and arthritis. Psychol Res Behav Manag.

[34] Berding A, Witte C, Gottschald M, Kaltz B, Weiland R, Gerlich C (2017). Beneficial effects of education on emotional distress, self-management, and coping in patients with inflammatory bowel disease: a prospective randomized controlled study. Inflamm.

[35] Reusch A, Weiland R, Gerlich C, Dreger K, Derra C, Mainos D (2016). Self-management education for rehabilitation inpatients suffering from inflammatory bowel disease: a cluster-randomized controlled trial. Health Educ Res.

[36] Oxelmark L, Magnusson A, Lofberg R, Hilleras P (2007). Group-based intervention program in inflammatory bowel disease patients: effects on quality of life. Inflamm Bowel Dis.

[37] Oliveira S, Zaltman C, Elia C, Vargens R, Leal A, Barros R (2007). Quality-of-life measurement in patients with inflammatory bowel disease receiving social support. Inflamm Bowel Dis.

[38] McDonnell E, Forry M, O'Raghallaigh JW, Kelly O, Patchett S, Ruane A (2014). Pilot study of a multitiered psychosocial support framework for inflammatory bowel disease patients. Gastrointestinal Nurs.

[39] Krause M (2003). The transformation of social representations of chronic disease in a self-help group. J Health Psychol.

[40] Haapamaki J, Heikkinen E, Sipponen T, Roine RP, Arkkila P (2018). The impact of an adaptation course on health-related quality of life and functional capacity of patients with inflammatory bowel disease. Scand J Gastroenterol.

[41] Shepanski MA, Hurd LB, Culton K, Markowitz JE, Mamula P, Baldassano RN (2005). Health-related quality of life improves in children and adolescents with inflammatory bowel disease after attending a camp sponsored by the Crohn's and Colitis Foundation of America. Inflamm Bowel Dis.

[42] Plevinsky JM, Greenley RN (2014). Exploring health-related quality of life and social functioning in adolescents with inflammatory bowel diseases after attending camp oasis and participating in a Facebook group. Inflamm Bowel Dis.

[43] Szigethy E, Hardy D, Craig AE, Low C, Kukic S (2009). Girls connect: effects of a support group for teenage girls with inflammatory bowel disease and their mothers. Inflamm Bowel Dis.

[44] Arenas DG, Pujol G, Mairena MA, Gutierrez A, Egea N, Termes M (2018). Results of a psychological and nutritional group program in pediatric patients with inflammatory bowel disease. J Pediatr Gastroenterol Nutr.

[45] Day A, McCombie A, Gearry RB (2016). Impact of an IBD camp upon disease-specific knowledge and quality of life in children and adolescents with inflammatory bowel disease. J Pediatr Gastroenterol Nutr.

[46] Hashash JG, Sigal R, Wein-Levy P, Szigethy EM, Merusi JJ, Regueiro MD (2016). Inflammatory bowel disease (IBD) connect: a novel volunteer program for hospitalized patients with IBD and their families. Inflamm Bowel Dis.

[47] Forry M, McDonnell E, Wilson O'Raghallaigh J, Kelly O, O'Toole A, Patchett S (2013). Examination of the efficacy of a chronic disease self-managementramme (CDSMP) for patients with inflammatory bowel disease (IBD): a pilot study. Gut.

[48] Forry M, McDonnell E, Wilson-O'Raghallaigh J, Kelly O, O'Toole A, Murray F (2013). Examination of the efficacy of Chronic Disease Self-management Programme (CDSMP) for patients with inflammatory bowel disease (IBD): a pilot study. J Crohn's Colitis.

[49] Plevinsky JM (2012). Continuing camp oasis: adolescent utilization of facebook for social support [M.A.].

[50] Regueiro M, Sigal R, Wein-Levy P, Hashash JG, Szigethy E (2016). IBD connect: a novel volunteer program for hospitalized crohn's disease and ulcerative colitis patients that improves stress and communication. Gastroenterology.

[51] Bartholomew Eldredge LK, Markham CM, Ruiter RA, Fernandez ME, Kok G, Parcel GS (2016). Planning health promotion programs: an intervention mapping approach.

[52] Larsson K, Sundberg Hjelm M, Karlbom U, Nordin K, Anderberg UM, Loof L (2003). A group-based patient education programme for high-anxiety patients with Crohn disease or ulcerative colitis. Scand J Gastroenterol.

[53] Gillard S, Gibson S, Holley J, Lucock M (2015). Developing a change model for peer worker interventions in mental health services: a qualitative research study. Epidemiol Psychiatr Sci.

[54] Gandek B, Ware JE, Aaronson NK, Apolone G, Bjorner JB, Brazier JE (1998). Cross-validation of item selection and scoring for the SF-12 Health Survey in nine countries: results from the IQOLA Project. International Quality of Life Assessment. J Clin Epidemiol.

[55] Thompson DM, Booth L, Moore D, Mathers J (2022). Peer support for people with chronic conditions: a systematic review of reviews. BMC Health Serv Res.

[56] Blockman B, Acree M, Becker D, Nichols A, Schaffer-White A, Winkelman M (2018). Effectiveness of a mind-body and peer support program for teens living with chronic illness and their parents: a pilot study. Glob Adv Health Med.

[57] Luu T, Hoogendoorn C, Armstrong K, Tabak B, Reigada L (2011). Peer support for adults with IBD: implications for quality of life and health practices. Inflamm Bowel Dis.

[58] Nct (2019). Peer i-coaching for activated self-management optimization in adolescents and young adults with chronic conditions.

[59] Nct (2019). Peer mentoring to improve self-management in youth with IBD.

[60] Nct (2018). iPeer2Peer program for adolescents with inflammatory bowel disease.

