# Supplementary material for: Peer support for carers and patients with inflammatory bowel disease: a systematic review
Source: Syst Rev. 2022 Sep 12;11:200. doi: 10.1186/s13643-022-02064-6 (PMC9465919; doi:10.1186/s13643-022-02064-6)
Supplement: Supplementary file 1 — Additional file 1: Supplementary Table 1. PRISMA 2020 Checklist. Supplementary Table 2. Search strategy for MEDLINE. Supplementary Table 3. Excluded studies and reason for exclusion. Supplementary Table 4. Risk of bias assessment. Supplementary Table 5. Health-related quality of life (HRQoL) in RCTs and non-randomised studies. Supplementary Table 6. Health-related quality of life (HRQoL) for before-and-after studies. Supplementary Table 7. Anxiety and depression in RCTs and non-randomised studies. Supplementary Table 8. Anxiety and depression in before-and-after studies. Supplementary Table 9. Patient education and knowledge about the illness. Supplementary Table 10. Other outcomes measured in RCTs. Supplementary Table 11. Other outcomes measured in before-and-after studies. [file 13643_2022_2064_MOESM1_ESM.docx]

Supplementary Table 1: PRISMA 2020 Checklist

| **Section and Topic** | **Item #** | **Checklist item** | **Location where item is reported** |
| --- | --- | --- | --- |
| **TITLE** | | |  |
| Title | 1 | Identify the report as a systematic review. | Cover page |
| **ABSTRACT** | | |  |
| Abstract | 2 | See the PRISMA 2020 for Abstracts checklist. | Cover page |
| **INTRODUCTION** | | |  |
| Rationale | 3 | Describe the rationale for the review in the context of existing knowledge. | Introduction section |
| Objectives | 4 | Provide an explicit statement of the objective(s) or question(s) the review addresses. | Introduction, final paragraph |
| **METHODS** | | |  |
| Eligibility criteria | 5 | Specify the inclusion and exclusion criteria for the review and how studies were grouped for the syntheses. | Methods > Selection Criteria and Study Selection |
| Information sources | 6 | Specify all databases, registers, websites, organisations, reference lists and other sources searched or consulted to identify studies. Specify the date when each source was last searched or consulted. | Methods > Search Strategy |
| Search strategy | 7 | Present the full search strategies for all databases, registers and websites, including any filters and limits used. | Appendices |
| Selection process | 8 | Specify the methods used to decide whether a study met the inclusion criteria of the review, including how many reviewers screened each record and each report retrieved, whether they worked independently, and if applicable, details of automation tools used in the process. | Methods > Selection Criteria and Study Selection |
| Data collection process | 9 | Specify the methods used to collect data from reports, including how many reviewers collected data from each report, whether they worked independently, any processes for obtaining or confirming data from study investigators, and if applicable, details of automation tools used in the process. | Methods > Data Extraction |
| Data items | 10a | List and define all outcomes for which data were sought. Specify whether all results that were compatible with each outcome domain in each study were sought (e.g. for all measures, time points, analyses), and if not, the methods used to decide which results to collect. | Methods > Data Extraction |
|  | 10b | List and define all other variables for which data were sought (e.g. participant and intervention characteristics, funding sources). Describe any assumptions made about any missing or unclear information. | Methods > Data Extraction |
| Study risk of bias assessment | 11 | Specify the methods used to assess risk of bias in the included studies, including details of the tool(s) used, how many reviewers assessed each study and whether they worked independently, and if applicable, details of automation tools used in the process. | Methods > Risk of Bias Assessment |
| Effect measures | 12 | Specify for each outcome the effect measure(s) (e.g. risk ratio, mean difference) used in the synthesis or presentation of results. | Methods > Analysis |
| Synthesis methods | 13a | Describe the processes used to decide which studies were eligible for each synthesis (e.g. tabulating the study intervention characteristics and comparing against the planned groups for each synthesis (item #5)). | N/A |
|  | 13b | Describe any methods required to prepare the data for presentation or synthesis, such as handling of missing summary statistics, or data conversions. | Methods > Data Extraction |
|  | 13c | Describe any methods used to tabulate or visually display results of individual studies and syntheses. | Methods > Analysis |
|  | 13d | Describe any methods used to synthesize results and provide a rationale for the choice(s). If meta-analysis was performed, describe the model(s), method(s) to identify the presence and extent of statistical heterogeneity, and software package(s) used. | i) Methods > Analysis  ii) Results > Risk of Bias Assessment |
|  | 13e | Describe any methods used to explore possible causes of heterogeneity among study results (e.g. subgroup analysis, meta-regression). | N/A |
|  | 13f | Describe any sensitivity analyses conducted to assess robustness of the synthesized results. | N/A |
| Reporting bias assessment | 14 | Describe any methods used to assess risk of bias due to missing results in a synthesis (arising from reporting biases). | Methods > Data Extraction |
| Certainty assessment | 15 | Describe any methods used to assess certainty (or confidence) in the body of evidence for an outcome. | N/A |
| **RESULTS** | | |  |
| Study selection | 16a | Describe the results of the search and selection process, from the number of records identified in the search to the number of studies included in the review, ideally using a flow diagram. | i) Results, paragraph 1-2  ii) Figure 1 |
|  | 16b | Cite studies that might appear to meet the inclusion criteria, but which were excluded, and explain why they were excluded. | Appendices |
| Study characteristics | 17 | Cite each included study and present its characteristics. | i) Results > first four subsections  ii) Table 1-2 |
| Risk of bias in studies | 18 | Present assessments of risk of bias for each included study. | i) Results > Risk of bias Assessment  ii) Appendices |
| Results of individual studies | 19 | For all outcomes, present, for each study: (a) summary statistics for each group (where appropriate) and (b) an effect estimate and its precision (e.g. confidence/credible interval), ideally using structured tables or plots. | i) Results > Effectiveness of Peer Support Interventions  ii) Appendices |
| Results of syntheses | 20a | For each synthesis, briefly summarise the characteristics and risk of bias among contributing studies. | i) Results > Risk of bias Assessment  ii) Appendices |
|  | 20b | Present results of all statistical syntheses conducted. If meta-analysis was done, present for each the summary estimate and its precision (e.g. confidence/credible interval) and measures of statistical heterogeneity. If comparing groups, describe the direction of the effect. | N/A |
|  | 20c | Present results of all investigations of possible causes of heterogeneity among study results. | Results > Risk of Bias Assessment |
|  | 20d | Present results of all sensitivity analyses conducted to assess the robustness of the synthesized results. | N/A |
| Reporting biases | 21 | Present assessments of risk of bias due to missing results (arising from reporting biases) for each synthesis assessed. | N/A |
| Certainty of evidence | 22 | Present assessments of certainty (or confidence) in the body of evidence for each outcome assessed. | N/A |
| **DISCUSSION** | | |  |
| Discussion | 23a | Provide a general interpretation of the results in the context of other evidence. | Discussion > |
|  | 23b | Discuss any limitations of the evidence included in the review. | Discussion > Strengths and limitations of the included studies |
|  | 23c | Discuss any limitations of the review processes used. | Discussion > Strengths and limitations of the review |
|  | 23d | Discuss implications of the results for practice, policy, and future research. | i) Discussion > Recommendation for future research  ii) Conclusion |
| **OTHER INFORMATION** | | |  |
| Registration and protocol | 24a | Provide registration information for the review, including register name and registration number, or state that the review was not registered. | Methods section, first paragraph |
|  | 24b | Indicate where the review protocol can be accessed, or state that a protocol was not prepared. | Methods section, first paragraph |
|  | 24c | Describe and explain any amendments to information provided at registration or in the protocol. | N/A |
| Support | 25 | Describe sources of financial or non-financial support for the review, and the role of the funders or sponsors in the review. | See post-manuscript ‘Funding’ |
| Competing interests | 26 | Declare any competing interests of review authors. | See post-manuscript ‘Competing interests’ |
| Availability of data, code and other materials | 27 | Report which of the following are publicly available and where they can be found: template data collection forms; data extracted from included studies; data used for all analyses; analytic code; any other materials used in the review. | Appendices |

*From:*  Page MJ, McKenzie JE, Bossuyt PM, Boutron I, Hoffmann TC, Mulrow CD, et al. The PRISMA 2020 statement: an updated guideline for reporting systematic reviews. BMJ 2021;372:n71. doi: 10.1136/bmj.n71

For more information, visit: <http://www.prisma-statement.org/>

Supplementary Table 2: Search strategy for MEDLINE

| **MEDLINE(R) and In-Process & Other Non-Indexed Citations (via Ovid)** |
| --- |
| 1. (peer adj2 (group$ or support$ or intervention$ or forum or fora or workshop$ or session$ or meet$ or network$ or counsel$ or advice or advis$ or communit$ or visit$ or social$ or promot$)).ti,ab. 2. (support$ adj2 (peer$ or group$ or intervention$ or forum or fora or workshop$ or session$ or meet$ or network$ or counsel$ or advice or advis$ or communit$ or visit$ or colleague$ or family or families or friend$)).ti,ab. 3. (support$ adj3 (mHealth or mobile or SMS or text messag$ or Telehealth or eHealth or Health information technolog$ or Internet)).ti,ab. 4. exp Peer group/ 5. exp Telemedicine/ and support$.ti,ab. 6. (community adj2 (network$ or involve$ or cohesion or building or develop$)).ti,ab. 7. (social adj (capital or relationship$)).ti,ab. 8. exp Self-Management/ 9. self-management.ti,ab. 10. Self care/ 11. exp Social Support/ 12. exp Self-help groups/ 13. self-help.ti,ab. 14. exp Mentors/ 15. exp Mentoring/ 16. (mentor$ or mentee$).ti,ab. 17. (one on one or one to one or face to face).ti,ab. 18. Patient Education as Topic/ 19. patient education.ti,ab. 20. IBD Connect.ti,ab. 21. budd$.ti,ab. 22. or/1-21 23. exp Inflammatory bowel disease/ 24. inflammatory bowel disease$.ti,ab. 25. IBD.ti,ab. 26. exp Colitis, Ulcerative/ 27. colitis.ti,ab. 28. exp Crohn disease/ 29. Crohn$.ti,ab. 30. pancolitis.ti,ab. 31. proctitis.ti,ab. 32. proctocolitis.ti,ab. 33. or/23-32 34. 22 and 33 |

| **EMBASE** |
| --- |
| 1. (peer adj2 (group$ or support$ or intervention$ or forum or fora or workshop$ or session$ or meet$ or network$ or counsel$ or advice or advis$ or communit$ or visit$ or social$ or promot$)).ti,ab. 2. (support$ adj2 (peer$ or group$ or intervention$ or forum or fora or workshop$ or session$ or meet$ or network$ or counsel$ or advice or advis$ or communit$ or visit$ or colleague$ or family or families or friend$)).ti,ab. 3. (support$ adj3 (mHealth or mobile or SMS or text messag$ or Telehealth or eHealth or Health information technolog$ or Internet)).ti,ab. 4. exp Peer support/ 5. exp Telemedicine/ and support$.ti,ab. 6. (community adj2 (network$ or involve$ or cohesion or building or develop$)).ti,ab. 7. (social adj (capital or relationship$)).ti,ab. 8. exp Self-Management/ 9. self-management.ti,ab. 10. Self care/ 11. exp Social Support/ 12. exp Support Group/ 13. exp Self help/ 14. self-help.ti,ab. 15. exp Mentors/ 16. exp Mentoring/ 17. (mentor$ or mentee$).ti,ab. 18. (one on one or one to one or face to face).ti,ab. 19. Exp Patient Education/ 20. patient education.ti,ab. 21. IBD Connect.ti,ab. 22. budd$.ti,ab. 23. or/1-22 24. exp Inflammatory bowel disease/ 25. inflammatory bowel disease$.ti,ab. 26. IBD.ti,ab. 27. Exp Ulcerative Colitis/ 28. colitis.ti,ab. 29. Exp Crohn disease/ 30. Crohn$.ti,ab. 31. pancolitis.ti,ab. 32. proctitis.ti,ab. 33. proctocolitis.ti,ab. 34. or/24-33 35. 23 and 34 |

| **The Cochrane Library (Cochrane Central Register of Controlled Trials (CENTRAL)** |
| --- |
| 1. peer NEAR/2 (group* or support* or intervention* or forum or fora or workshop* or session* or meet* or network* or counsel* or advice or advis* or communit* or visit* or social* or promot*):ti,ab, kw 2. support* NEAR/2 (peer* or group* or intervention* or forum or fora or workshop* or session* or meet* or network* or counsel* or advice or advis* or communit* or visit* or colleague* or family or families or friend*):ti,ab, kw 3. support* NEAR/3 (mHealth or mobile or SMS or text-messag* or Telehealth or eHealth or “Health information technology” or “Health information technologies” or Internet):ti,ab, kw 4. MeSH descriptor: [Peer Group] explode all trees 5. MeSH descriptor: [Telemedicine] explode all trees and support*:ti,ab, kw 6. community NEAR/2 (network* or involve* or cohesion or building or develop*):ti,ab, kw 7. social NEXT (capital or relationship*):ti,ab, kw 8. MeSH descriptor: [Self-Management] explode all trees 9. self-management:ti,ab, kw 10. MeSH descriptor: [Self Care] this term only 11. MeSH descriptor: [Social Support] explode all trees 12. MeSH descriptor: [Self-help groups] explode all trees 13. self-help:ti,ab, kw 14. MeSH descriptor: [Mentors] explode all trees 15. MeSH descriptor: [Mentoring] explode all trees 16. (mentor* or mentee*):ti,ab, kw 17. (“one on one” or one-on-one or “one to one” or one-to-one or “face to face” or face-to-face):ti,ab, kw 18. MeSH descriptor: [Patient Education as Topic] 19. “patient education”:ti,ab, kw 20. IBD Connect :ti,ab, kw. 21. budd*:ti,ab, kw 22. or/1-21 23. MeSH descriptor: [inflammatory bowel diseases] explode all trees 24. inflammatory bowel disease*:ti,ab, kw 25. IBD:ti,ab, kw 26. MeSH descriptor: [Colitis, Ulcerative] explode all trees 27. colitis:ti,ab, kw 28. MeSH descriptor: [Crohn Disease] explode all trees 29. Crohn*:ti,ab, kw 30. pancolitis:ti,ab, kw 31. proctitis :ti,ab, kw 32. proctocolitis:ti,ab, kw 33. or/23-32 34. 22 and 33 |

| **PSYCINFO** |
| --- |
| 1. (peer adj2 (group$ or support$ or intervention$ or forum or fora or workshop$ or session$ or meet$ or network$ or counsel$ or advice or advis$ or communit$ or visit$ or social$ or promot$)).ti,ab. 2. (support$ adj2 (peer$ or group$ or intervention$ or forum or fora or workshop$ or session$ or meet$ or network$ or counsel$ or advice or advis$ or communit$ or visit$ or colleague$ or family or families or friend$)).ti,ab. 3. (support$ adj3 (mHealth or mobile or SMS or text-messag$ or Telehealth or eHealth or Health information technolog$ or Internet)).ti,ab. 4. exp Peers/ 5. exp Telemedicine/ and support$.ti,ab. 6. (community adj2 (network$ or involve$ or cohesion or building or develop$)).ti,ab. 7. (social adj (capital or relationship$)).ti,ab. 8. exp Self-Management/ 9. self-management.ti,ab. 10. Self-care skills/ 11. exp Social Support/ 12. exp Support Groups/ 13. self-help.ti,ab. 14. exp Mentor/ 15. (mentor$ or mentee$).ti,ab. 16. (one on one or one to one or face to face).ti,ab. 17. patient education.ti,ab. 18. IBD Connect.ti,ab. 19. budd$.ti,ab. 20. or/1-19 21. inflammatory bowel disease$.ti,ab. 22. IBD.ti,ab. 23. exp Ulcerative Colitis/ 24. colitis.ti,ab. 25. Crohn$.ti,ab. 26. pancolitis.ti,ab. 27. proctitis.ti,ab. 28. proctocolitis.ti,ab. 29. or/21-28 30. 20 and 29 |

| **CINAHL** |
| --- |
| 1. TI ( (peer n2 (group* or support* or intervention* or forum or fora or workshop* or session* or meet* or network* or counsel* or advice or advis* or communit* or visit* or social* or promot*)) ) OR AB ( (peer n2 (group* or support* or intervention* or forum or fora or workshop* or session* or meet* or network* or counsel* or advice or advis* or communit* or visit* or social* or promot*)) ) 2. TI ( (support* n2 (peer* or group* or intervention* or forum or fora or workshop* or session* or meet* or network* or counsel* or advice or advis* or communit* or visit* or colleague* or family or families or friend*)) ) OR AB ( (support* n2 (peer* or group* or intervention* or forum or fora or workshop* or session* or meet* or network* or counsel* or advice or advis* or communit* or visit* or colleague* or family or families or friend*)) ) 3. TI ( (support* n3 (mHealth or mobile or SMS or text-messag* or Telehealth or eHealth or “Health information technology” or “Health information technologies” or Internet)) ) OR AB ( (support* n3 (mHealth or mobile or SMS or text messag* or Telehealth or eHealth or “Health information technology” or “Health information technologies” or Internet)) ) 4. MH “Peer Group” 5. MH "Telemedicine+" AND (TI support* OR AB support*) 6. TI ( (community n2 (network* or involve* or cohesion or building or develop*)) ) OR AB ( (community n2 (network* or involve* or cohesion or building or develop*)) ) 7. TI ( (social n (capital or relationship*)) ) OR AB ( (social n (capital or relationship*)) 8. MH “Self-Management” 9. TI ( self-management or “self management” ) OR AB ( self-management or “self management” ) 10. MH “Self Care” 11. MH "Caregiver Support" 12. MH "Support Groups+" 13. TI ( “self help” or self-help ) OR AB ( “self help” or self-help ) 14. MH "Mentorship" 15. TI ( mentor* or mentee* ) OR AB ( mentor* or mentee* ) 16. TI (“one on one” or “one-on-one” or “one to one” or “one-to-one” or “face to face”) OR AB (“one on one” or “one-on-one” or “one to one” or “one-to-one” or “face to face”) 17. MH "Patient Education+" 18. TI “patient education” OR AB “patient education” 19. TI IBD Connect OR AB IBD Connect 20. TI budd* OR AB budd* 21. or/1-20 22. MH "Inflammatory Bowel Diseases+" 23. TI ( inflammatory bowel disease* ) OR AB ( inflammatory bowel disease* ) 24. TI ( IBD ) OR AB ( IBD ) 25. MH "Colitis, Ulcerative" 26. TI ( colitis ) OR AB ( colitis ) 27. MH “Crohn disease” 28. TI ( crohn* ) OR AB ( crohn* ) 29. TI ( pancolitis ) OR AB ( pancolitis ) 30. TI ( proctitis ) OR AB ( proctitis ) 31. TI ( proctocolitis ) OR AB ( proctocolitis ) 32. or/22-31 33. 21 AND 32 |

| **Conference Proceedings Citation Index (via Web of Science)** |
| --- |
| Search field: Topic  (peer* or support* or budd* or mentor* or mentee* or "self-management" or "self management" or "self help" or "self-help" or " patient education") AND (inflammatory bowel disease or IBD or ulcerative colitis or crohn or proctitis or proctocolitis or pancolitis) |

| **PROQUEST** |
| --- |
| (AB, TI(peer* or support* or budd* or mentor* or mentee* or "self-management" or "self management" or "self help" or "self-help" or" patient education") or su(peer) or su(self help) or su(selfmanagement) or su(social support) or su(patient education)) AND (AB,TI(inflammatory bowel disease or IBD or ulcerative colitis or crohn or proctitis or proctocolitis or pancolitis) OR su(inflammatory bowel disease) OR su(ulcerative colitis) OR su(crohn disease)) |

| **OPEN GREY** |
| --- |
| (peer* OR support* OR budd* OR mentor* OR mentee* OR self-management OR self management OR self help OR self-help OR patient education) AND (inflammatory bowel disease OR IBD OR ulcerative colitis OR crohn OR proctitis OR proctocolitis OR pancolitis) |

| **WHO ICTRP** |
| --- |
| Inflammatory bowel disease OR IBD OR ulcerative colitis OR crohn’s disease OR proctitis OR proctocolitis OR pancolitis  AND  Peer OR support OR buddies OR mentor OR self-help OR self-management OR patient education OR mHealth OR eHealth |

| **Clinicaltrials.gov** |
| --- |
| Inflammatory bowel disease OR IBD OR ulcerative colitis OR crohn’s disease OR proctitis OR proctocolitis OR pancolitis  AND  Peer OR support OR buddies OR mentor OR self-help OR self-management OR patient education |

| **International Standard Randomised Controlled Trials Number** |
| --- |
| Condition: Inflammatory bowel disease  Condition: Crohn's disease  Condition: ulcerative colitis |

Supplementary Table 3: Excluded studies and reason for exclusion

| STUDY ID | REASON FOR EXCLUSION |
| --- | --- |
|  | **Intervention not including peer support** |
| Ahola 2019 | Mindfulness-based intervention incorporating mindfulness meditations and exercises |
| Atreja 2017 | Patient education app |
| Atreja 2017 (2) | Patient education app |
| Atreja 2018 | Patient education app |
| Bascom 2017 | Study evaluating perceived peer support |
| Bokemeyer 2016 | Complex intervention with individualized recommendations for appropriate treatment, implementation of interdisciplinary IBD case conferences and offer of a group-based patient education program |
| Egon 2017 | Individual and collective educational sessions |
| Greenley 2015 | Phone-based problem solving skills training sessions between families and graduate students |
| Grootenhuis 2009 | Psychoeducational intervention focusing on training on information giving and seeking |
| Gupta 2006 | Yoga and relaxation techniques |
| Jaghult 2007 | Group-based education programme |
| Johnson 2018 | Online support via an IBD web-portal |
| Langhorst 2007 | Lifestyle modification programme |
| Larsson 2003 | Patient education programme |
| Maddux 2017 | Adherence-promoting intervention provided to patients individually |
| Maunder 2001 | Supportive-expressive group psychotherapy |
| Meng 2018 | Self-management education programme |
| Moradkhani 2012 | Study evaluating unspecified support groups |
| Moskovitz 2000 | Study evaluating community resources in general |
| Nikolaus 2014 | Patient education programme |
| Nikolaus 2014 (2) | Patient education programme |
| On 2017 | IBD education clinic |
| Szigethy 2017 | Mobile cognitive-based therapy |
| Tripp 2017 | Cognitive-behavioural self-management programme |
|  | **Outcomes are not relevant** |
| Clarke 2016 | Patient satisfaction |
| Fearn 2019 | Domain of support received by couches |
| Fearn 2019 (2) | Social media usage metrics |
| Keefer 2018 | GRITT score to quantify individual’s strengths |
| Mayberry 1987 | Desired future activities of the group |
| McMaster 2012 | Client-rated satisfaction, perceived social support and qualitative outcomes |
| Miguel 2014 | Patients discussing their disease with family and friends |
| Rezailashkajani 2008 | Number of website visitors, content evaluation and readability |
| Takacs 1994 | Patient satisfaction in relation to the support group |
|  | **Ineligible study design** |
| Bernick 2010 | Review |
| Blaskovich 2012 | Presentation given to a support group to address the clinical features of the diseases and ways to be an active participant in care |
| Brotkin 2016 | Presentation of peer coaching programme |
| Donegan 2016 | Article about implementation of parent mentoring programme |
| Garcia 2002 | It is a commentary |
| Lichtenstein 2009 | It is a letter |
| Luttenberg 1996 | Article about author’s experience |
| Messner 1984 | Article about author’s experience |
| Morohoshi 2012 | Lifestyle guidance |
| Nagahori 2012 | Guidance on social support |
| Pullen 1998 | Description of disease and treatments |
| Salazar 2014 | Qualitative study |
| Stewart 2014 | Mixed-method study |
| Strohmeyer 2006 | Article about nurse’s experience with support groups |
| Waldecker 1988 | It is a commentary |
| Zigron 2019 | Qualitative study |
|  | **Other** |
| Beasley 2016 | Educational and psychosocial support to children and adolescents with IBD but it is not clear whether it entails peer support (conference abstract) |
| Nelson 2018 | Mobile videoconferencing-delivered support group intervention but it is not clear whether it entails peer support (conference abstract) |
| Tongiorgi 2016 | Psychological support groups but it is not clear whether it entails peer support (conference abstract) |
| Vettorato 2016 | Patient support programme but it is not clear whether it entails peer support (conference abstract) |
|  | **Full-text not available** |
| Driscoll 1997 | Full-text could not be obtained |
| Haslbeck 1993 | Full-text could not be obtained |
| Myer 1978 | Full-text could not be obtained |

**Search update excluded studies and reason for exclusion**

| STUDY ID | REASON FOR EXCLUSION |
| --- | --- |
|  | **Ineligible Population** |
| Malori 2020 | Not IBD |
|  | **Intervention not including peer support** |
| Huppe 2020 | Inpatient rehabilitation without peer support |
| Mickoka-Wallis 2020 | Expressive writing without peer support |
| Otilia 2019 | No clear indication of peer support as part of intervention |
|  | **Ineligible study design** |
| Gates 2021 | Development and feasibility only |

Supplementary Table 4: Risk of bias assessment

Risk of bias in controlled studies

| Study ID | Risk of bias judgement | | | | | |
| --- | --- | --- | --- | --- | --- | --- |
|  | *Selection Bias*  *Random sequence generation* | *Selection Bias*  *Allocation concealment* | *Performance bias*  *Blinding of participants and personnel* | *Detection bias*  *Blinding of outcome assessment* | *Attrition bias*  *Incomplete outcome data* | *Reporting Bias*  *Selective outcome reporting* |
| Randomised controlled trials | | | | | | |
| Berding 2017^(34)^ | *Low*  Participants randomised using computer-generated numbers. Block-randomisation method was used. | *Low*  Participants randomised through central randomisation, randomisation numbers conveyed by telephone or e-mail. | *High*  Participants could not be blinded. The lack of blinding may have influenced their performance when answering the questionnaires. | High  Outcomes are patient-reported and participants could not be blinded. The lack of blinding may have influenced their responses to the questionnaires. | *Low*  Attrition greater in intervention group (18% vs 6.8%). A sensitivity analysis revealed no differences between the analytic sample and the participants who dropped out. | *Unclear*  No access to study protocol |
| Oliveira 2007^(37)^ | *Unclear*  Patients randomised- no further details are provided | *Unclear*  The method of concealment is not described. | *High*  Participants could not be blinded. The lack of blinding may have affected their performance when answering the questionnaires. | *High*  Outcomes are patient-reported and participants could not be blinded. The lack of blinding may have influenced their responses to the questionnaires. | *High*  40% of participants in intervention group did not complete the study. This may have generated systematic differences between the two groups. | *High*  No access to study protocol. Results from SF-36 questionnaire have not been reported. |
| Oxelmark 2007^(36)^ | *Unclear*  Patients randomised- no further details are provided. | *Unclear*  Method of concealment is not described. | *High*  Participants could not be blinded. The lack of blinding may have affected their performance when answering the questionnaires. | *High*  Outcomes are patient-reported and participants could not be blinded. The lack of blinding may have influenced their responses to the questionnaires. | *Low*  Reason for missing data is unknown. Similar percentage of LTFU /missing data between the two groups (17-25% in the intervention group vs 25% in the control group). Last value carried forward analysis was performed and showed no differences between the two groups. | *Unclear*  No access to study protocol. |
| Reusch 2016^(35)^ | *Unclear*  Patients randomised- no further details are provided. | *Low*  Randomisation performed by telephone. | *High*  Participants could not be blinded. The lack of blinding may have affected their performance when answering the questionnaires. | *High*  Outcomes are patient-reported and participants could not be blinded. The lack of blinding may have influenced their responses to the questionnaires. | *Unclear*  18% did not return data at 3 months. It is stated participants who dropped out were older, more often not living with a partner and more likely to suffer from CD, and no differences were evident for gender and education. However, data are not shown. | *Unclear*  Data provided at 3 months but the outcomes have also been measured at 12 months. |
| Zhang 2020^(33)^ | *Low*  Patients randomised- via random number table. | *Unclear*  The method of concealment is not described. | *High*  Participants could not be blinded. The lack of blinding may have affected their performance when answering the questionnaires. | High  Outcomes are patient-reported and participants could not be blinded. The lack of blinding may have influenced their responses to the questionnaires. | *Low*  Attrition equal across each group (9%). Differences between the analytic sample and the participants who dropped out were not reported. | *Unclear*  No access to study protocol. |
| Non-randomised controlled trials | | | | | | |
| Krause 2003^(39)^ | *Not applicable*  Participants are not randomised. The two groups are comparable according to author but systematic differences between the two groups are possible. | *Not applicable*  Not clear how people have been recruited to the control group. The two groups are comparable according to author but they seem to come from different source. | *High*  Participants could not be blinded. The lack of blinding may have affected their performance when answering the questionnaires. | *High*  Outcomes are patient-reported and participants could not be blinded. The lack of blinding may have influenced their responses to the questionnaires. | *Unclear*  It seems that there are no missing data, no clear information is reported. | *Unclear*  No access to study protocol |
| McDonnell 2014^(38)^ | *Not applicable*  Participants were not randomised. Sample of convenience were created and it is likely groups were recruited from same population. | *Not applicable*  Participants were not randomised. Sample of convenience were created. | *High*  Participants could not be blinded. The lack of blinding may have affected their performance when answering the questionnaires. | *High*  Outcomes are patient-reported and participants could not be blinded. The lack of blinding may have influenced their responses to the questionnaires. | *High*  33% and 50% of participants in the intervention and control group, respectively, did not return the questionnaire at 3 months. The reasons have not been clarified. | *Unclear*  No access to study protocol |

Risk of bias table for before-and-after studies

| DOMAIN | RISK OF BIAS JUDGEMENT | | | | |
| --- | --- | --- | --- | --- | --- |
|  | Haapamäki 2018^(40)^ | Hashash 2016^(46)^ | Plevinsky 2014^(42)^ | Shepanski 2005^(41)^ | Szigethy 2009^(43)^ |
| Was the study question or objective clearly stated? | *Yes*  Objectives clearly stated | *Yes*  Objectives clearly stated | *Yes*  Objectives clearly stated | *Yes*  Clear question stated | Yes  Objectives are stated |
| Were eligibility/selection criteria for the study population pre-specified and clearly described? | *No*  Eligibility criteria are very broad. | NR  Eligibility criteria not reported | *Yes*  Eligibility criteria specified | *NR*  Eligibility criteria not reported | *CD*  Eligibility criteria not clearly described but seem broad and inclusive |
| Were the participants in the study representative of those who would be eligible for the test/service/intervention in the general or clinical population of interest? Were all eligible participants that met the pre-specified entry criteria enrolled? | *CD*  People participating in adaptation courses may have more concerns about their disease or they may have a more complicated disease course compared to an average person with IBD. | *CD*  Participants’ characteristics not reported in detail. | *CD*  Half of the participants had attended a camp before (i.e. they may have already benefited from the camp). The sample was above average in their Facebook use characteristics to the general population of adolescents. Of the 89 campers contacted, 25 (31%) signed up to participate. | *CD*  People who do not actively participate in the CCFA were not recruited, they may be different from those who do. | *Yes*  Eligibility criteria not clearly described but seem broad and inclusive. The intervention was meant for girls and mothers only. |
| Was the sample size sufficiently large to provide confidence in the findings? | *CD*  Data are provided for 142 participants. | *Yes*  The sample size is large (677 participants). | *CD*  Data provided for 21 participants. Effect size analyses were emphasized over traditional statistical significance testing as the power to detect statistically significant differences was expected to be quite low with a small sample size. | *CD*  Data provided for 61 participants (55 for anxiety outcomes). | *CD*  Data provided for 11 participants. |
| Was the test/service/intervention clearly described and delivered consistently across the study population? | *No*  Different adaptation courses were tested and were probably adapted to each patient situation. | *Yes*  Intervention described in details. | *Yes*  It is likely the intervention was delivered consistently across the study population | *Yes*  Volunteers were trained, it is likely intervention was delivered consistently across all study participants. | *No*  Not every participant attended all sessions; this may have generated heterogeneity in the type of education topic and number of sessions attended. |
| Were the outcome measures pre-specified, clearly defined, valid, reliable, and assessed consistently across all study participants? | Yes  Outcomes are pre-specified.  Tools have been chosen based on evidence in literature. | *No*  Outcomes are specified. Questions were not validated as this was not performed as part of a formal research protocol. | *Yes*  Outcomes clearly defined, references are reported for instrument validity and reliability. | *Yes*  Outcomes are clearly defined. Volunteers were trained in how to administer the questionnaires. | *CD*  Outcomes are not stated explicitly in the introductory part of the letter.  It is likely questionnaires were administered consistently across all study participants. |
| Were the people assessing the outcomes blinded to the participants' exposures/interventions? | *No*  Outcomes are self-reported by participants who are not blinded (no controlled group). The lack of blinding may have impacted their responses in the questionnaires. | *No*  Outcomes are self-reported by participants who are not blinded (no controlled group). The lack of blinding may have impacted their responses in the questionnaires. | *No*  Outcomes are self-reported by participants who are not blinded (no controlled group). The lack of blinding may have impacted their responses in the questionnaires. | *No*  Outcomes are self-reported by participants who are not blinded (no controlled group). The lack of blinding may have impacted their responses in the questionnaires. | *No*  Outcomes are self-reported by participants who are not blinded (no controlled group). The lack of blinding may have impacted their responses in the questionnaires. |
| Was the loss to follow-up after baseline 20% or less? Were those lost to follow-up accounted for in the analysis? | *No*  27% did not return the follow-up questionnaires. Not clear whether these participants were different from those who completed the questionnaires. | *No*  77/677 (11.4%) responded to the survey. | *Yes*  16% of the participants did not complete the questionnaire but it is stated that the start of the school year might have made them busier. | *No*  51% did not return the questionnaire and were not included in the analysis. | *Yes*  92% completed questionnaires at both time points. |
| Did the statistical methods examine changes in outcome measures from before to after the intervention? | *Yes*  Changes in outcomes were measured but p-values are reported only. | *Yes*  Only p-values provided, not the change from baseline. | *Yes*  Mean change was calculated. | *Yes*  Repeated measures t-test was employed | *CD*  Unpaired t-test was employed. |

Abbreviations: CD (cannot determine), NA (not applicable), NR (not reported)

Risk of bias table for before-and-after studies (conference abstracts)

| DOMAIN | RISK OF BIAS JUDGEMENT | |
| --- | --- | --- |
|  | Arenas 2018^(44)^ | Day 2016^(45)^ |
| Was the study question or objective clearly stated? | *Yes*  Objectives clearly stated | *Yes*  Objectives clearly stated |
| Were eligibility/selection criteria for the study population pre-specified and clearly described? | *NR*  Eligibility criteria not reported | *NR*  Eligibility criteria not reported |
| Were the participants in the study representative of those who would be eligible for the test/service/intervention in the general or clinical population of interest? Were all eligible participants that met the pre-specified entry criteria enrolled? | *No*  Study sample too small to allow reasonable degree of specificity in representativeness. Neither eligibility criteria nor participants’ characteristics are reported. | *Yes*  64% of the participants only were in remission. This may imply that eligibility criteria were inclusive in terms of disease activity |
| Was the sample size sufficiently large to provide confidence in the findings? | *No*  Very small sample size (8 people). It is unlikely that small significant differences could be detected. | *CD*  Data provided for 39 participants. |
| Was the test/service/intervention clearly described and delivered consistently across the study population? | *Yes*  Main components of the intervention are described and, being a group-based intervention, it can be assumed it was delivered consistently across all study participants. | *NR*  Not detailed information is provided. |
| Were the outcome measures pre-specified, clearly defined, valid, reliable, and assessed consistently across all study participants? | *NR*  Tools used are reported but not in great details. | *Yes*  Outcomes are pre-specified. Campers were asked to complete questionnaires, likely they were consistently administered across group. |
| Were the people assessing the outcomes blinded to the participants' exposures/interventions? | *No*  Outcomes are self-reported by participants who are not blinded (there is no controlled group). The lack of blinding may have impacted their responses in the questionnaires. | *No*  Outcomes are self-reported by participants who are not blinded (there is no controlled group). The lack of blinding may have impacted their responses in the questionnaires. |
| Was the loss to follow-up after baseline 20% or less? Were those lost to follow-up accounted for in the analysis? | *NR*  No details are reported. | *No*  23% and 35% of participants did not provide the questionnaires at 1 and 6 months, respectively.  *NR*  Not clear whether LTFU were accounted for in the analysis. |
| Did the statistical methods examine changes in outcome measures from before to after the intervention? | *NR*  No details are reported. | *Yes*  Comparisons were made using the paired Student’s t test. |

Abbreviations: CD (cannot determine), NA (not applicable), NR (not reported)

Supplementary Table 5: Health-related quality of life (HRQoL) in RCTs and non-randomised studies

| Study | Tool | Scale  (worst-best) | Time points | N of participants | Mean (SD) Intervention group | N of participants | Mean (SD) control group | Between-group mean difference (95%CI)  ≤3 months | Between-group mean difference (95%CI)  >3 months and ≤1 year | Between-group mean difference (95%CI)  >1 year |
| --- | --- | --- | --- | --- | --- | --- | --- | --- | --- | --- |
| RCTs |  |  |  |  |  |  |  |  |  |  |
| Berding 2017(34) | SF-12   Physical domain | 0-100 | Baseline | 86 | 46.58 (9.20) | 95 | 45.99 (8.79) |  |  |  |
|  |  |  | 3 months | 84 | 47.62 (9.08) | 95 | 46.60 (9.16) | p=0.54 |  |  |
|  | SF-12  Mental domain | 0-100 | Baseline | 86 | 44.31 (11.76) | 95 | 42.74 (10.37) |  |  |  |
|  |  |  | 3 months | 84 | 46.41 (11.00) | 95 | 42.70 (10.89) | p=0.18 |  |  |
| Reusch  2016(35) | SF-12  Physical domain | 0-100 | Baseline | 252 | 41.78 (10.00) | 264 | 39.85 (10.85) |  |  |  |
|  |  |  | 3 months | 252 | 43.57 (10.24) | 264 | 44.09 (10.22) | p=0.06 |  |  |
|  | SF-12  Mental domain | 0-100 | Baseline | 252 | 36.46 (11.40) | 264 | 37.15 (11.14) |  |  |  |
|  |  |  | 3 months | 252 | 41.81 (11.85) | 264 | 40.80 (12.03) | p=0.13 |  |  |
| Oxelmark 2007(36) | IBDQ  Disease-specific  (with score for single domains) | 32-224 | Baseline | 24 | Total 173.9 (28.0) | 20 | 182.8 (30.2) |  |  |  |
|  |  |  |  |  | Bowel 54.1 (9.4) |  | 57.6 (11.6) |  |  |  |
|  |  |  |  |  | Systemic 25.6 (5.5) |  | 27.6 (4.4) |  |  |  |
|  |  |  |  |  | Emotional  63.6 (11.7) |  | 65.0 (13.7) |  |  |  |
|  |  |  |  |  | Social 30.6 (5.8) |  | 32.6 (3.1) |  |  |  |
|  |  |  | 6 months | 18 | Total 175.7 (35.0) | 15 | 187.9 (27.7) |  | p>0.20 |  |
|  |  |  |  |  | Bowel 55.1 (11.2) |  | 58.2 (11.2) |  | p>0.20 |  |
|  |  |  |  |  | Systemic 26.1 (6.2) |  | 27.2 (5.4) |  | p>0.20 |  |
|  |  |  |  |  | Emotional  64.6 (12.5) |  | 69.8 (10.3) |  | p>0.20 |  |
|  |  |  |  |  | Social 29.8 (7.7) |  | 32.7 (3.9) |  | p>0.20 |  |
|  |  |  | 12 months | 20 | Total 171.8 (28.2) | 15 | 173.7 (28.2) |  | p>0.20 |  |
|  |  |  |  |  | Bowel 56.1 (9.0) |  | 56.0 (12.0) |  | p>0.20 |  |
|  |  |  |  |  | Systemic 25.1 (5.3) |  | 26.1 (5.5) |  | p>0.20 |  |
|  |  |  |  |  | Emotional  65.7 (11.5) |  | 66.8 (10.8) |  | p>0.20 |  |
|  |  |  |  |  | Social 30.9 (4.8) |  | 30.3 (4.7) |  | p>0.20 |  |
| Oliveira 2007(37) | IBDQ  Disease-specific (with score for single domains) | 32-224 | Baseline | 16 | Total  151.81 (35.23) | 12 | NR |  |  |  |
|  |  |  |  |  | Bowel  51.88 (11.12) |  |  |  |  |  |
|  |  |  |  |  | Systemic  22.13 (5.75) |  |  |  |  |  |
|  |  |  |  |  | Emotional  54.56 (16.37) |  |  |  |  |  |
|  |  |  |  |  | Social  23.25 (8.40) |  |  |  |  |  |
|  |  |  | 5-7 months | 16 | Total 165.19 (41.81) | 12 | NR |  | NR |  |
|  |  |  |  |  | Bowel  53.31 (11.85) |  |  |  |  |  |
|  |  |  |  |  | Systemic  24.25 (7.18) |  |  |  |  |  |
|  |  |  |  |  | Emotional  63.06 (18.94) |  |  |  |  |  |
|  |  |  |  |  | Social  24.56 (8.43) |  |  |  |  |  |
|  |  |  | 11-13 months | 16 | Total  161.25 (40.63) | 12 | NR |  | NR |  |
|  |  |  |  |  | Bowel  52.13 (12.15) |  |  |  |  |  |
|  |  |  |  |  | Systemic  22.69 (6.67) |  |  |  |  |  |
|  |  |  |  |  | Emotional  61.25 (17.91) |  |  |  |  |  |
|  |  |  |  |  | Social  25.19 (8.81) |  |  |  |  |  |
|  |  |  | 17-19 months | 16 | Total  170.88 (42.85) | 12 | NR |  |  | NR |
|  |  |  |  |  | Bowel  55.63 (12.68) |  |  |  |  |  |
|  |  |  |  |  | Systemic  23.81 (6.32) |  |  |  |  |  |
|  |  |  |  |  | Emotional  62.81 (20.35) |  |  |  |  |  |
|  |  |  |  |  | Social  28.69 (7.74) |  |  |  |  |  |
| Non-randomised studies | | |  |  |  |  |  |  |  |  |
| McDonnell 2014(38) | SIBDQ  Disease-specific | 10-70 | Baseline | 27 | NR | 26 | NR |  |  |  |
|  |  |  | 3 months | 18 | NR | 13 | 0.6 increase in mean score | 0.2 decrease in mean score |  |  |
|  | SF-36  General | 0-100 | Baseline | 27 | NR | 26 | NR |  |  |  |
|  |  |  | 3 months | 18 | NR | 13 | NR | Improvements in intervention group compared to control group at a statistically significant level on 2 domains (General Health Scale and Energy/Fatigue) |  |  |
| Krause 2007(39) | SIBDQ  Disease-specific  (with score for single domains) | 10-70 | Baseline | 19 | NR | 19 | NR |  |  |  |
|  |  |  | End of intervention (1 year) | 19 | Total 45.05 (14.65) | 19 | 49.35 (13.06) |  | p=0.276 |  |
|  |  |  |  |  | Social 10.89 (3.91) |  | 10.41 (3.73) |  | p=0.659 |  |
|  |  |  |  |  | Emotional  12.05 (4.48) |  | 14.09 (4.16) |  | p=0.103 |  |
|  |  |  |  |  | Intestinal  12.42 (5.77) |  | 15.74 (4.84) |  | p=0.030 |  |

Abbreviations: CI (confidence interval), (S)IBDQ ((Short) IBD Questionnaire), NR (not reported), SD (standard deviation), SF-12 (Short Form- 12 items), SF-36 (Short Form- 36 items)

Supplementary Table 6: Health-related quality of life (HRQoL) for before-and-after studies

| Study | Tool | Scale  (worst-best) | Time points | N of participants | Mean (SD) Intervention group | Within-group  Difference (95%CI)  ≤3 months | Within-group  Difference (95%CI)  >3 months and ≤1 year |
| --- | --- | --- | --- | --- | --- | --- | --- |
| Szigethy 2009(43) | IMPACT-III  Disease-specific | 35-175 | Baseline | 11 | 82.64 (27.08) |  |  |
|  |  |  | End of programme (10 months) | 11 | 67.92 (13.24) |  | p<0.10 |
| Haapamaki 2018(40) | 15-D  General | 0-1 | Baseline | 142 | 0.816 |  |  |
|  |  |  | end of course | 142 | 0.837 | 0.014 [0.007, 0.022] |  |
|  |  |  | 6 months | 142 | 0.853 |  | 0.033 [0.021, 0.045] |
|  |  |  | 12 months | 142 | 0.854 |  | 0.027 [0.015, 0.040] |
| Shepanski 2005(41) | IMPACT-II  Disease-specific | 0-245 | Baseline | 61 | 172.95 (36.61) |  |  |
|  |  |  | 1 week (postcamp) | 61 | 178.71 (40.97) | 5.7 [0.52, 10.88] |  |
| Plevinsky 2014(42) | IMPACT-III  Disease-specific | 35-175 | Baseline | 21 | 128.80 (21.76) |  |  |
|  |  |  | 1 week (postcamp) | 21 | 136.89 (20.23) | 8.09 [3.24, 12.93] |  |
|  |  |  | 2 months (since participation in Facebook group) | 21 | 132.10 (18.85) | **Baseline/Post-Facebook**:  3.29 [-3.16, 9.74]  **Postcamp/post-Facebook**:  4.81 [-2.36, 11.98] |  |
| Day 2016(45) | IMPACT III  Disease-specific | 35-175 | Baseline | 39 | 133.83 (20.79) |  |  |
|  |  |  | 1 month | 30 | 135.2 (23.39) | p=0.8117 |  |
|  |  |  | 6 months | 25 | 137.9 (20.7) |  | p=0.581 |

Abbreviations: CI (confidence interval), SD (standard deviation)

Supplementary Table 7: Anxiety and depression in RCTs and non-randomised studies

| Study | Tool | Scale  (worst-best) | Time points | N of participants | Mean (SD) Intervention group | N of participants | Mean (SD)  Control group | Between-group  mean difference (95%CI)  ≤3 months | Between-group mean difference (95%CI)  >3 months and ≤1 year |
| --- | --- | --- | --- | --- | --- | --- | --- | --- | --- |
| RCTs |  |  |  |  |  |  |  |  |  |
| Berding 2017(34) | PHQ-4  Anxiety | 6-0 | Baseline | 86 | 1.64 (1.38) | 95 | 1.73 (1.44) |  |  |
|  |  |  | 2 weeks | 84 | 1.48 (1.38) | 95 | 1.65 (1.38) | p=0.56 |  |
|  |  |  | 3 months | 84 | 1.46 (1.47) | 95 | 1.57 (1.47) | p=0.75 |  |
|  | PHQ-4  Depression | 6-0 | Baseline | 86 | 1.71 (1.49) | 95 | 1.82 (1.49) |  |  |
|  |  |  | 2 weeks | 84 | 1.36 (1.21) | 95 | 1.72 (1.43) | p=0.11 |  |
|  |  |  | 3 months | 84 | 1.48 (1.40) | 95 | 1.65 (1.45) | p=0.63 |  |
| Reusch  2016(35) | GAD-2  Anxiety | 6-0 | Baseline | 252 | 2.47 (1.64) | 264 | 2.63 (1.77) |  |  |
|  |  |  | 3 months | 252 | 2.00 (1.63) | 264 | 2.08 (1.61) | p=0.93 |  |
|  | PHQ-2  Depression | 6-0 | Baseline | 252 | 2.45 (1.67) | 264 | 2.72 (1.63) |  |  |
|  |  |  | 3 months | 252 | 2.15 (1.67) | 264 | 2.09 (1.61) | p=0.18 |  |
| Zhang 2020_(33)_ | HADS-d  Depression | 21-0 | Baseline | 30 | 15.59 (4.25)* | 30 | 15.12 (2.86)* |  |  |
|  |  |  | 6 weeks‡ | 30 | 10.31 (3.23)* | 30 | 13.01 (3.02)* | p<0.05† |  |
| Non-randomised studies | | |  |  |  |  |  |  |  |
| McDonnell 2014(38) | HADS scale  Anxiety | 21-0 | Baseline | 27 | 22% | 26 | 38% |  |  |
|  |  |  | 3 months | 18 | 4% | 13 | 27% | NR  No significant effect for depression |  |

*Median (Interquartile range); † Peers support only vs Routine treatment control; ‡ Zhang also reports measurement was taken 1 month after intervention

Abbreviations: CI (confidence interval), GAD-2 (Generalised Anxiety Disorder 2-item), HADS (Hospital Anxiety and Depression Scale), NR (not reported), PHQ (Patient Health Questionnaire), SD (standard deviation)

Supplementary Table 8: Anxiety and depression in before-and-after studies

| Study | Instrument used | Scale  (worst-best) | Time points | N of participants | Mean (SD) Intervention group | Within-group  Difference (95%CI)  ≤3 months | Within-group  Difference (95%CI)  >3 months and ≤1 year |
| --- | --- | --- | --- | --- | --- | --- | --- |
| Haapamaki 2018(40) | BDI  Depression | 63-0 | Baseline | 142 | 11.8 |  |  |
|  |  |  | End of course | 142 | 8.5 | 2.853 [2.178, 3.528] |  |
|  |  |  | 6 months | 142 | 8.9 |  | 2.765 [1.577, 3.953] |
|  |  |  | 12 months | 142 | 9.3 |  | 2.118 [1.030, 3.206] |
| Arenas 2018(44) | STAI  Anxiety | NR | Baseline | 8 | NR |  |  |
|  |  |  | 6 weeks (end of programme) | 8 | NR | Anxiety levels were reduced of 50% |  |
| Shepanski 2005(41) | STAIC  Anxiety | 120-40 | Baseline | 55 | NR |  |  |
|  |  |  | 1 week (postcamp) | 55 | NR | P>0.05  (both state and trait anxiety scales) |  |

Abbreviations: BDI (Beck's Depression Inventory), CI (confidence interval), NR (not reported), SD (standard deviation), STAI(C) (State-Trait Anxiety Inventory (Children))

Supplementary Table 9: Patient education and knowledge about the illness

| Study | Instrument used | Scale  (worst-best) | Time points | N of participants | Mean (SD) Intervention group | N of participants | Mean (SD)  Control group | Between-group  mean difference (95%CI)  ≤3 months | Within-group difference (95%CI) |
| --- | --- | --- | --- | --- | --- | --- | --- | --- | --- |
| RCTs |  |  |  |  |  |  |  |  |  |
| Berding 2017(34) | In-house/  **Level of knowledge** |  |  | | | | | | |
|  | a) Medical aspects |  | Baseline  2 weeks  3 months | 86  84  84 | 3.35 (0.72)  4.23 (0.48)  4.05 (0.41) | 95  95  95 | 3.42 (0.74)  3.44 (0.65)  3.42 (0.71) | p<0.001  p<0.001 |  |
|  | b) Psychological aspects |  | Baseline  2 weeks  3 months | 86  84  84 | 2.84 (0.97)  3.81 (0.72)  3.65 (0.67) | 95  95  95 | 2.85 (0.88)  2.99 (0.70)  2.98 (0.74) | p<0.001  p<0.001 |  |
|  | HeiQ/  **Patient education impact** |  |  | | | | | | |
|  | a) Constructive attitudes and approaches |  | Baseline  2 weeks  3 months | 86  84  84 | 3.25 (0.53)  3.38 (0.50)  3.34 (0.48) | 95  95  95 | 3.17 (0.51)  3.12 (0.55)  3.10 (0.59) | p<0.001  p=0.001 |  |
|  | b) Skills and techniques acquisition |  | Baseline  2 weeks  3 months | 86  84  84 | 2.80 (0.51)  3.06 (0.48)  3.08 (0.41) | 95  95  95 | 2.62 (0.63)  2.66 (0.54)  2.67 (0.53) | p<0.001  p<0.001 |  |
|  | c) Positive and active engagement in life |  | Baseline  2 weeks  3 months | 86  84  84 | 3.09 (0.54)  3.19 (0.54)  3.16 (0.49) | 95  95  95 | 2.99 (0.57)  3.04 (0.56)  2.98 (0.59) | p=0.20  p=0.10 |  |
| Reusch  2016(35) | In-house/  **Level of knowledge** |  |  | | | | | | |
|  | a) Medical aspects |  | Baseline  3 months | 252  252 | 3.20 (0.82)  3.93 (0.56) | 264  264 | 3.12 (0.89)  3.86 (0.65) | p=0.41 |  |
|  | b) Psychological aspects |  | Baseline  3 months | 252  252 | 2.47 (0.89)  3.58 (0.75) | 264  264 | 2.30 (0.98)  3.42 (0.82) | p=0.12 |  |
|  | HeiQ/  **Patient education impact** |  |  | | | | | | |
|  | a) Constructive attitudes and approaches |  | Baseline  3 months | 252  252 | 2.98 (0.63)  3.06 (0.61) | 264  264 | 2.94 (0.59)  3.08 (0.62) | p=0.49 |  |
|  | b) Skills and techniques acquisition |  | Baseline  3 months | 252  252 | 2.62 (0.58)  2.97 (0.55) | 264  264 | 2.59 (0.53)  2.92 (0.52) | p=0.57 |  |
|  | c) Positive and active engagement in life |  | Baseline  3 months | 252  252 | 2.79 (0.59)  2.87 (0.62) | 264  264 | 2.78 (0.59)  2.85 (0.58) | p=0.68 |  |
| Non-randomised studies | | |  |  |  |  |  |  |  |
| Krause 2003(39) | Questionnaire on knowledge about the illness |  | Baseline | 19 | 25.14 (5.31) | 19 | 25.41 (6.11) |  |  |
|  |  |  | End of intervention (1 year) | 19 | 27.28 (5.20) |  | NR |  | Intervention group  2.14 [0.64,3.6]  p=0.012 |
| Before-and-after studies | |  |  |  |  |  |  |  |  |
| Day 2016(45) | IBD-KID |  | Baseline | 39 | 10.67 (4.1) | NA | NA | NA |  |
|  |  |  | 1 month | 31 | 12.1 (3.4) | NA | NA | NA | p=0.032 |
|  |  |  | 6 months | 28 | 12.0 (4.1) | NA | NA | NA | p=0.040 |
| Haapamäki 2018(40) | Good knowledge of disease (%) | NA | Baseline | 142 | % of participants  73.4 | NA | NA | NA |  |
|  |  |  | End of course | 142 | 97.6 | NA | NA | NA | p=0.002 |
|  |  |  | 6 months | 142 | 98.9 | NA | NA | NA | p=0.001 |
|  |  |  | 12 months | 142 | 97.8 | NA | NA | NA | p=0.017 |

Abbreviations: CI (confidence interval); HeiQ (Health Education Impact Questionnaire), NA (not applicable)

Supplementary Table 10: Other outcomes measured in RCTs

| Study | Instrument used | Scale  (worst-best) | Time points | N of participants | Mean (SD) Intervention group | N of participants | Mean (SD) control group | Between-group mean difference (95%CI)  ≤3 months | Between-group mean difference (95%CI)  >3 months and ≤1 year |
| --- | --- | --- | --- | --- | --- | --- | --- | --- | --- |
| Disease activity | | | | | | | | | |
| Berding 2017(34) | GIBDI | 21-0 | Baseline | 86 | 2.84 (2.12) | 95 | 3.56 (2.50) |  |  |
|  |  |  | 2 weeks | 84 | 2.89 (2.36) | 95 | 3.64 (2.28) | p=0.65 |  |
|  |  |  | 3 months | 84 | 3.04 (2.77) | 95 | 3.76 (2.53) | p=0.57 |  |
| IBD patient concerns | | | | | | | | | |
| Berding 2017(34) | IBDPC (German version) |  | Baseline | 86 | 2.61 (0.74) | 95 | 2.63 (0.82) |  |  |
|  |  |  | 2 weeks | 84 | 2.26 (0.70) | 95 | 2.57 (0.83) | p<0.001 |  |
|  |  |  | 3 months | 84 | 2.25 (0.71) | 95 | 2.55 (0.85) | p<0.001 |  |
| Reusch 2016(35) | IBDPC  (German version) |  | Baseline | 252 | 2.66 (0.81) | 264 | 2.75 (0.83) |  |  |
|  |  |  | 3 months | 252 | 2.38 (0.81) | 264 | 2.50 (0.89) | p=0.30 |  |
| Fear of progression | | | | | | | | | |
| Berding 2017(34) | FoP-Q-SF |  | Baseline | 86 | 2.94 (0.66) | 95 | 2.78 (0.77) |  |  |
|  |  |  | 2 weeks | 84 | 2.64 (0.67) | 95 | 2.80 (0.74) | p<0.001 |  |
|  |  |  | 3 months | 84 | 2.63 (0.68) | 95 | 2.70 (0.74) | p=0.001 |  |
| Reusch 2016(35) | FoP-Q-SF |  | Baseline | 252 | 3.04 (0.75) | 264 | 3.07 (0.83) |  |  |
|  |  |  | 3 months | 252 | 2.76 (0.79) | 264 | 2.85 (0.84) | p=0.17 |  |
| Coping with anxiety | | | | | | | | | |
| Berding 2017(34) | FoP-Q |  | Baseline | 86 | 3.62 (0.57) | 95 | 3.35 (0.65) |  |  |
|  |  |  | 2 weeks | 84 | 3.68 (0.53) | 95 | 3.30 (0.59) | p=0.001 |  |
|  |  |  | 3 months | 84 | 3.70 (0.57) | 95 | 3.29 (0.66) | p=0.001 |  |
| Reusch 2016(35) | FoP-Q |  | Baseline | 252 | 3.26 (0.62) | 264 | 3.25 (0.58) |  |  |
|  |  |  | 3 months | 252 | 3.38 (0.69) | 264 | 3.33 (0.60) | p=0.24 |  |
| Coping | | | | | | | | | |
| Berding 2017(34) | In-house  With IBD | NR | Baseline  2 weeks  3 months | 86  84  84 | 3.70 (0.69) 4.08 (0.55)  4.06 (0.61) | 95  95  95 | 3.51 (0.77) 3.59 (0.75) 3.51 (0.73) | p<0.001  p<0.001 |  |
|  | With negative emotions |  | Baseline  2 weeks  3 months | 86  84  84 | 3.16 (0.97) 3.58 (0.78)  3.54 (0.81) | 95  95  95 | 3.00 (0.84) 3.19 (0.78) 3.18 (0.90) | p=0.001  p= 0.008 |  |
|  | Number of strategies |  | Baseline  2 weeks  3 months | 86  84  84 | 8.69 (3.35) 9.45 (3.50)  9.39 (3.58) | 95  95  95 | 8.06 (3.37) 8.27 (3.54) 8.03 (3.32) | p=0.05  p=0.011 |  |
| Sense of coherence | | | | | | | | | |
| Oxelmark 2007(36) | SOC questionnaire | 29-203 | Baseline | 24 | 139.4 (21.7) | 20 | 147.6 (20.6) |  |  |
|  |  |  | 6 months | 18 | 137.6 (21.9) | 15 | 149.3 (23.1) |  | p=0.14 |
|  |  |  | 12 months | 20 | 137.3 (23.8) | 15 | 145.6 (26.5) |  | p=0.14 |
| Pain |  |  |  |  |  |  |  |  |  |
| Zhang 2020_(33)_ | Numerical rating scale | 10-0 | Baseline | 30 | 6.01 (1.86)* | 30 | 5.56 (1.18)* |  |  |
|  |  |  | 6 weeks‡ | 30 | 1.09 (0.76)* | 30 | 1.13 (0.26)* | p<0.05† |  |
| Sleep |  |  |  |  |  |  |  |  |  |
| Zhang 2020_(33)_ | Polysomnography | Sleep efficiency ratio | Baseline | 30 | 72.17 (13.01)* | 30 | 73.28 (12.25)* |  |  |
|  |  |  | 6 weeks‡ | 30 | 74.45 (11.92)* | 30 | 73.55 (10.98)* | p<0.05† |  |
| Irritable bowel syndrome | |  |  |  |  |  |  |  |  |
| Zhang 2020_(33)_ | Medical record review | No (n) | Baseline | 30 | 18 | 30 | 15 |  |  |
|  |  |  | 6 weeks‡ | 30 | 16 | 30 | 13 | p<0.05† |  |
| Inflammatory factor 6 | | |  |  |  |  |  |  |  |
| Zhang 2020_(33)_ | Enzyme labeling instrument |  | Baseline | 30 | 148.44 (15.27)* | 30 | 152.32 (20.24)* |  |  |
|  |  |  | 6 weeks‡ | 30 | 52.5 (12.37)* | 30 | 55.28 (12.61)* | p>0.05† |  |
| Inflammatory factor 17 | | |  |  |  |  |  |  |  |
| Zhang 2020_(33)_ | Enzyme labeling instrument |  | Baseline | 30 | 324.44 (25.12)* | 30 | 335.38 (22.21)* |  |  |
|  |  |  | 6 weeks‡ | 30 | 301.33 (16.35)* | 30 | 305.45 (17.69)* | p>0.05† |  |
| Inflammatory factor 23 | | |  |  |  |  |  |  |  |
| Zhang 2020_(33)_ | Enzyme labeling instrument |  | Baseline | 30 | 606.28 (40.76)* | 30 | 621.88 (34.56)* |  |  |
|  |  |  | 6 weeks‡ | 30 | 367.34 (33.25)* | 30 | 398.54 (28.67)* | p<0.05† |  |
| Inflammatory factor 35 | | |  |  |  |  |  |  |  |
| Zhang 2020_(33)_ | Enzyme labeling instrument |  | Baseline | 30 | 48.11 (15.54)* | 30 | 46.76 (11.63)* |  |  |
|  |  |  | 6 weeks‡ | 30 | 49.45 (15.67)* | 30 | 48.87 (15.43)* | p>0.05† |  |

*Median (Interquartile range); † Peers support only vs Routine treatment control; ‡ Zhang also reports measurement was taken 1 month after intervention

Supplementary Table 11: Other outcomes measured in before-and-after studies

| Study | Instrument used | Scale (worst-best) | Time points | N of participants | Mean (SD)  Intervention group | Within-group difference (95%CI)  ≤3 months | Within-group difference (95%CI)  >3 months and ≤ 1 year |
| --- | --- | --- | --- | --- | --- | --- | --- |
| Disease activity | |  |  |  |  |  |  |
| Haapamäki 2018(40) | HBI (CD)  Mayo score (UC) | NR  *HBI*  *>16 severe activity*  *Mayo score*  *12-0* | Baseline | 40 | HBI 4.0 |  |  |
|  |  |  |  | 95 | Mayo score 2.7 |  |  |
|  |  |  | 6 months | 40 | HBI 3.2 |  | 0.304 [-1.251, 1.860] |
|  |  |  |  | 95 | Mayo score 1.3 |  | 1.523 [1.061, 1.985] |
|  |  |  | 12 months | 40 | HBI 3.7 |  | 0.550 [-1.512, 2.612] |
|  |  |  |  | 95 | Mayo score 1.5 |  | 1.125 [0.561, 1.689] |
|  | **Health care services** | NA |  |  | Mean/median |  |  |
|  | a) Visits to health centre doctor or nurse |  | Baseline  6 months  12 months | 142  142  142 | 2.6/1.0  1.3/0.0  1.3/0.0 |  |  |
|  | b) Visits to gastroenterologist |  | Baseline  6 months  12 months | 142  142  142 | 0.6/0.0  0.7/0.0  0.5/0.0 |  |  |
|  | c) Laboratory tests |  | Baseline  6 months  12 months | 142  142  142 | 2.1/2.0  1.8/1.0  1.4/1.0 |  |  |
|  | d) Radiological examinations |  | Baseline  6 months  12 months | 142  142  142 | 0.2/0.0  0.2/0.0  0.2/0.0 |  |  |
|  | e) Endoscopies |  | Baseline  6 months  12 months | 142  142  142 | 0.4/0.0  0.2/0.0  0.2/0.0 |  |  |
|  | Sick-leave days |  | Baseline  6 months  12 months | 142  142  142 | 10.7/0.0  7.3/0.0  8.3/0.0 |  |  |
| Social Connectedness | | | | | |  | |
| Plevinsky 2014(42) | Revised Social Connectedness Questionnaire | 20-120 | Baseline | 21 | 93.00 (12.74) |  |  |
|  |  |  | 1 week (postcamp) | 21 | 96.12 (15.49) | 3.09 [-3.55, 9.73] |  |
|  |  |  | 2 months (since participation in Facebook group) | 21 | 96.14 (13.80) | 3.14 [-2.47, 8.75] |  |
| Social support | | | | | | | |
| Plevinsky 2014(42) | Social Support Questionnaire |  |  | | | | |
|  | a) Satisfaction^[[1]](#footnote-1)^ | 6-36 | Baseline  1 week (postcamp)  2 months (since participation in Facebook group) | 21  21  21 | 5.12 (1.04)  5.36 (0.75)  5.63 (0.64) | 0.24 [-0.26, 0.74]  0.48 [0.02, 0.94] |  |
|  | b) Number^[[2]](#footnote-2)^ | 10-54 | Baseline  1 week  2 months | 21  21  21 | 3.89 (1.68)  4.25 (1.58)  4.72 (1.94) | 0.24 [-0.16, 0.64]  0.62 [-0.30, 1.54] |  |
| Stress during hospitalisation | | | | | |  | |
| Hashash  2016(46) | In-house survey |  | Baseline | 77 | N of participants/percentage  44 (56%) |  |  |
|  |  |  | After IBD connect visit | 77 | 14 (18%) | p=0.001 |  |

Abbreviations: CD (Crohn’s disease), CI (confidence interval), FoP-Q-SF (Fear of Progression Questionnaire- Short Form), GIBDI (German Inflammatory Bowel Disease Activity Index ), HBI (Harvey-Bradshaw Index), IBDPC (IBD Patient Concerns), NA (not applicable), SD (standard deviation), SOC (Sense of Coherence), UC (ulcerative colitis)

1. Participants’ satisfaction with the support they receive. [↑](#footnote-ref-1)
2. Number of people who felt comfortable in accessing for social support [↑](#footnote-ref-2)
